# Nanoporous Anodic Alumina Photonic Crystals for Optical Chemo- and Biosensing: Fundamentals, Advances, and Perspectives

**DOI:** 10.3390/nano8100788

**Published:** 2018-10-04

**Authors:** Cheryl Suwen Law, Siew Yee Lim, Andrew D. Abell, Nicolas H. Voelcker, Abel Santos

**Affiliations:** 1 School of Chemical Engineering, The University of Adelaide, Adelaide SA 5005, Australia; suwen.law@adelaide.edu.au (C.S.L.); siew.lim@adelaide.edu.au (S.Y.L.); 2 Institute for Photonics and Advanced Sensing (IPAS), The University of Adelaide, Adelaide SA 5005, Australia; 3 ARC Centre of Excellence for Nanoscale BioPhotonics (CNBP), The University of Adelaide, Adelaide SA 5005, Australia; 4 Department of Chemistry, The University of Adelaide, Adelaide SA 5005, Australia; 5 Melbourne Centre for Nanofabrication, Victorian Node of the Australian National Fabrication Facility, Melbourne 3168, Australia; 6 Drug Delivery, Disposition and Dynamics, Monash Institute of Pharmaceutical Sciences, Monash University, Melbourne 3052, Australia; 7 Commonwealth Scientific and Industrial Research Organisation (CSIRO), Melbourne 3168, Australia; 8 INM-Leibniz Institute for New Materials, Campus D2 2, 66123 Saarbrücken, Germany

**Keywords:** optical sensing, photonic crystals, anodization, nanoporous anodic alumina, surface chemistry

## Abstract

Optical sensors are a class of devices that enable the identification and/or quantification of analyte molecules across multiple fields and disciplines such as environmental protection, medical diagnosis, security, food technology, biotechnology, and animal welfare. Nanoporous photonic crystal (PC) structures provide excellent platforms to develop such systems for a plethora of applications since these engineered materials enable precise and versatile control of light–matter interactions at the nanoscale. Nanoporous PCs provide both high sensitivity to monitor in real-time molecular binding events and a nanoporous matrix for selective immobilization of molecules of interest over increased surface areas. Nanoporous anodic alumina (NAA), a nanomaterial long envisaged as a PC, is an outstanding platform material to develop optical sensing systems in combination with multiple photonic technologies. Nanoporous anodic alumina photonic crystals (NAA-PCs) provide a versatile nanoporous structure that can be engineered in a multidimensional fashion to create unique PC sensing platforms such as Fabry–Pérot interferometers, distributed Bragg reflectors, gradient-index filters, optical microcavities, and others. The effective medium of NAA-PCs undergoes changes upon interactions with analyte molecules. These changes modify the NAA-PCs’ spectral fingerprints, which can be readily quantified to develop different sensing systems. This review introduces the fundamental development of NAA-PCs, compiling the most significant advances in the use of these optical materials for chemo- and biosensing applications, with a final prospective outlook about this exciting and dynamic field.

## 1. Introduction

Optical sensors are a class of devices that utilize different forms of light–matter (i.e., photon–atom) interactions to detect, interrogate, and quantify molecules for multiple applications. They contain a light source to generate electromagnetic waves, a sensing platform in which light–matter interactions occur, and a detector to identify and quantify spectral shifts in electromagnetic waves upon interaction and exposure to analytes [1,2,3]. Typically, the sensing principle in optical sensors relies on shifts in the characteristic spectral fingerprint of the optical platform upon interaction with analyte molecules. These spectral changes are subsequently translated into quantitative and/or qualitative measurements of molecules of interest. The design and engineering of the sensing platform is of paramount importance since this is where interactions between electromagnetic waves and analyte molecules occur [4,5]. Solid state optical sensing platforms can be produced in the form of thin films, fibers, and nanoparticles, which can guide, enhance, reflect, transmit, modulate, or absorb electromagnetic waves in different ways. The rapid development of nanotechnology has opened new opportunities and paths to develop optical sensing platforms with finely tuned optical properties. These platforms can be combined with spectroscopic techniques to produce outstanding sensing systems such as surface plasmon resonance spectroscopy (SPR) [4,5], localized surface plasmon resonance spectroscopy (LSPR) [6,7,8,9], surface enhanced Raman spectroscopy (SERS) [10,11], photoluminescence spectroscopy (PL) [12,13], and reflectometric interference spectroscopy (RIfS) [14,15].

Photonic crystals (PCs) are a type of optical materials that mold the flow of electromagnetic waves by multiple Bragg scattered interferences defined by Bloch modes [16,17,18]. Light propagation in PCs can be controlled with precision by engineering the structural features of the PCs in a multidimensional fashion (i.e., 1D, 2D, or 3D). Nanoporous PCs are particularly good candidates to develop ultra-sensitive optical sensing platforms since they provide (i) light-modifying capabilities to alter and engineer the flow of photons at specific broadband spectral regions (i.e., from UV to IR), (ii) a nanoporous structure that facilitates mass transport of molecular species involved in binding events, and (iii) high specific surface area that increases the number of functional binding sites within the optical platform [19]. Furthermore, nanoporous PCs can be produced by a range of cost-competitive, fully scalable self-organization approaches that provide excellent control over the PC’s features at the nanoscale. For example, inverted opal structures produced by a combination of self-organization of silica nanospheres and deposition of oxides or metals are one of the most representative types of nanoporous PCs [20,21]. However, these nanostructures have limited versatility to tune the photonic stopband (PSB) of PCs, are restricted to 3D nanostructures, feature defects that act as light scattering centers, require long synthesis processes (>24 h), and are constrained to small areas (mm^2^–cm^2^) [22]. Another prime example of nanoporous PC platform material is porous silicon (pSi), which is typically produced by electrochemical etching of silicon in hydrofluoric acid (HF)-based electrolytes [23,24,25]. Although, pSi presents excellent optoelectronic properties to develop optical sensing systems, its practical application is limited by its poor chemical stability without additional passivation steps, its fragile mechanical strength, and its fabrication process, which requires the use of extremely hazardous HF-based electrolytes [26,27]. Among other alternatives, nanoporous anodic alumina photonic crystals (NAA-PCs) produced by electrochemical oxidation (i.e., anodization) of aluminum—an industrially scalable and low-cost nanofabrication approach used in industry for decades—has been devised as an excellent alternative/complementary platform material to develop optical sensing systems in combination with a broad range of photonic technologies [28]. The nanoporous structure of NAA can be engineered by means of different anodization approaches to produce PC structures with finely tuned optical properties across the spectral regions. NAA provides controllable and versatile nanopore geometry, chemical and physical stability, stable and tunable optical signals, and mechanical strength. Furthermore, NAA’s surface chemistry can be modified with a broad range of functional molecules to achieve chemical selectivity toward analytes of interest [29].

**Figure 1:**
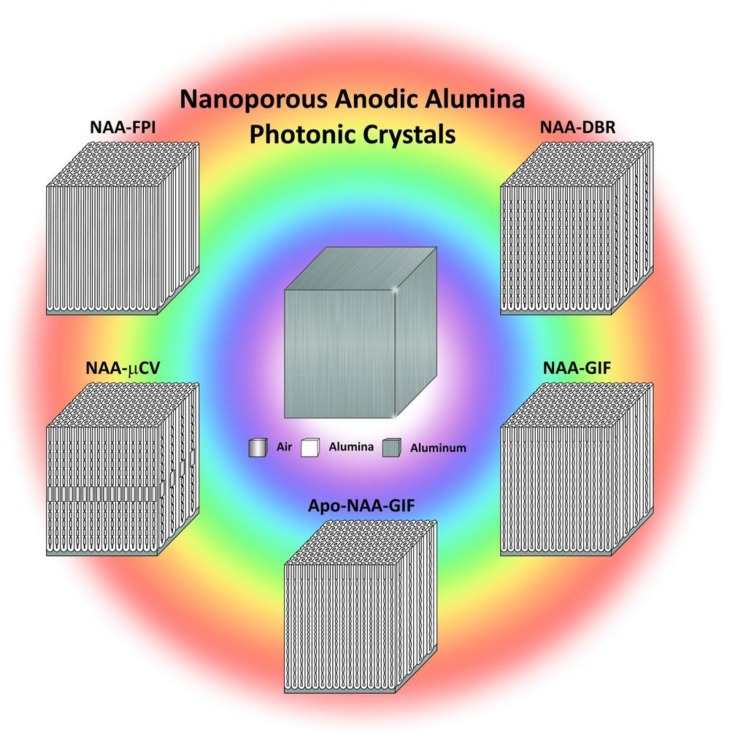
Nanoporous anodic alumina photonic crystals as effective medium platforms to control light–matter interactions for chemo- and biosensing applications. Schemes of representative NAA-PC structures: NAA-FPI—Fabry–Pérot interferometer; NAA-μCV—optical microcavity; NAA-APO-GIF—apodized gradient index filter; NAA-GIF—gradient index filter; NAA-DBR—distributed Bragg reflector.

Recent advances in anodization technology have focused on engineering the nanoporous structure of NAA to control optical properties and have paved the way for advanced NAA-PC-based sensing systems with promising performances and broad applicability (Figure 1). This review provides a comprehensive perspective about the fundamentals of NAA-PC technology, introducing the different fabrication processes and aspects of this singular PC platform material (i.e., chemical and physical properties, nanoporous structure and optical features, surface chemistry modification protocols, etc.) that make it an excellent candidate for optical chemo- and biosensing systems. The most significant advances in the development of NAA-PC-based optical sensing systems are commented upon in detail, with outstanding representative examples of applicability. Finally, this review concludes with a general overview and a prospective outlook on the future trends in this exciting and dynamic field.

## 2. Fabrication and Properties: Nanoporous Anodic Alumina as Effective Medium

### 2.1. Fabrication of Self-Organized Nanoporous Anodic Alumina

**Figure 2:**
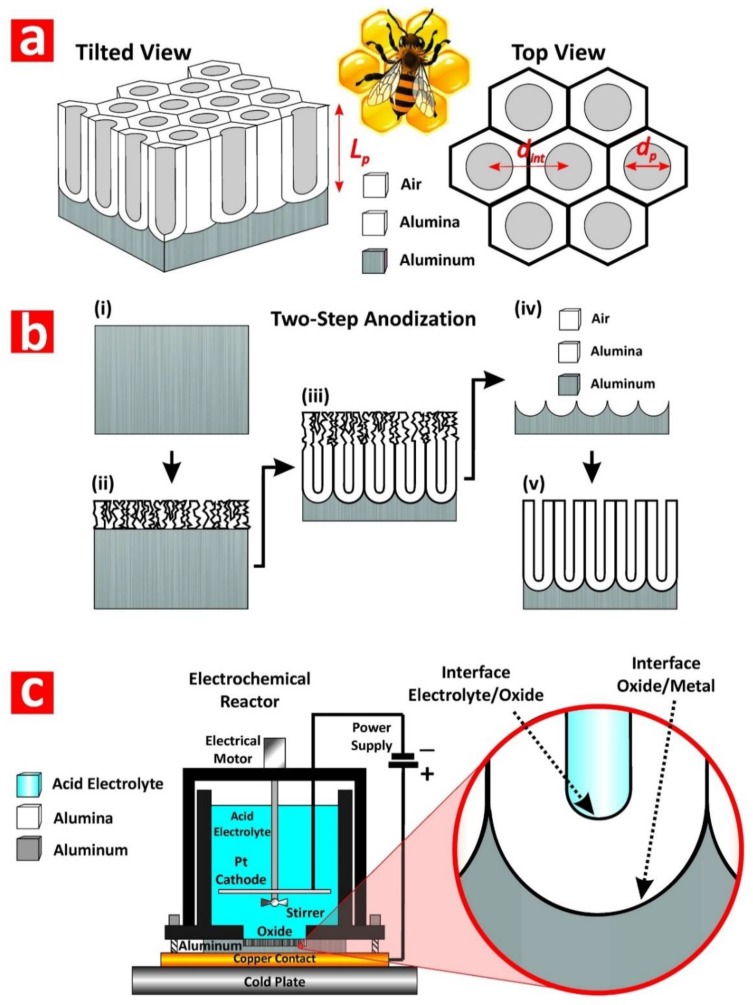
Self-organized nanoporous anodic alumina (NAA). (**a**) Structure and geometric features of NAA produced by two-step anodization with tilted 3D view of NAA structure (**left**) (*L_p_*—nanopores length) and top view of NAA structure (**right**) (*d_int_*—interpore distance and *d_p_*—nanopores diameter). (**b**) Schematic diagram illustrating the two-step anodization process: (**i**) aluminum substrate, (**ii**) nanopores nucleation—first step, (**iii**) nanopores development—first step, (**iv**) sacrificial oxide layer removal, and (**v**) cross-sectional view of self-organized NAA structure—second step. (**c**) Schematic illustration of an electrochemical reactor used to NAA by anodization (**left**) and details of the oxide barrier layer located at the bottom tip of the nanopores where the electrochemical reactions (i.e., oxidation and dissolution) occur at the interfaces metal/oxide and oxide/electrolyte.

Nanoporous anodic alumina (NAA) is a thin film composed of a matrix of alumina (aluminum oxide—Al_2_O_3_) featuring arrays of straight cylindrical nanopores with closed hemispherical bottom tips that grow at the center of hexagonal cells normally aligned to the underlying aluminum substrate (Figure 2a) [28]. Under specific fabrication conditions, the nanopores of NAA self-organize in a hexagonal fashion, leading to a characteristic honeycomb-like nanoporous structure with tuneable geometric features. NAA is produced by electrochemical oxidation of aluminum substrates in different mild acid electrolytes, the most representative of which are oxalic, sulfuric, and phosphoric acids [30,31,32]. This electrochemical process, so-called ‘anodization’, has been extensively and intensively used in metal finishing industry for more than a century. For example, anodization is used to modify the properties of valve metal parts and structures such as corrosion protection, adhesion, color and appearance, hardness, and impact and erosion resistance. However, despite its intensive use in industry, anodization of aluminum only gained significant attention in nanotechnology after the milestone works of Masuda and coworkers in the mid-1990s, with the introduction of the self-ordered NAA and the two-step anodization process [30,31,32]. These seminal works boosted an increasing and dynamic research activity in this field, spreading the applicability of these nanomaterials across disciplines and fields.

**Figure 3:**
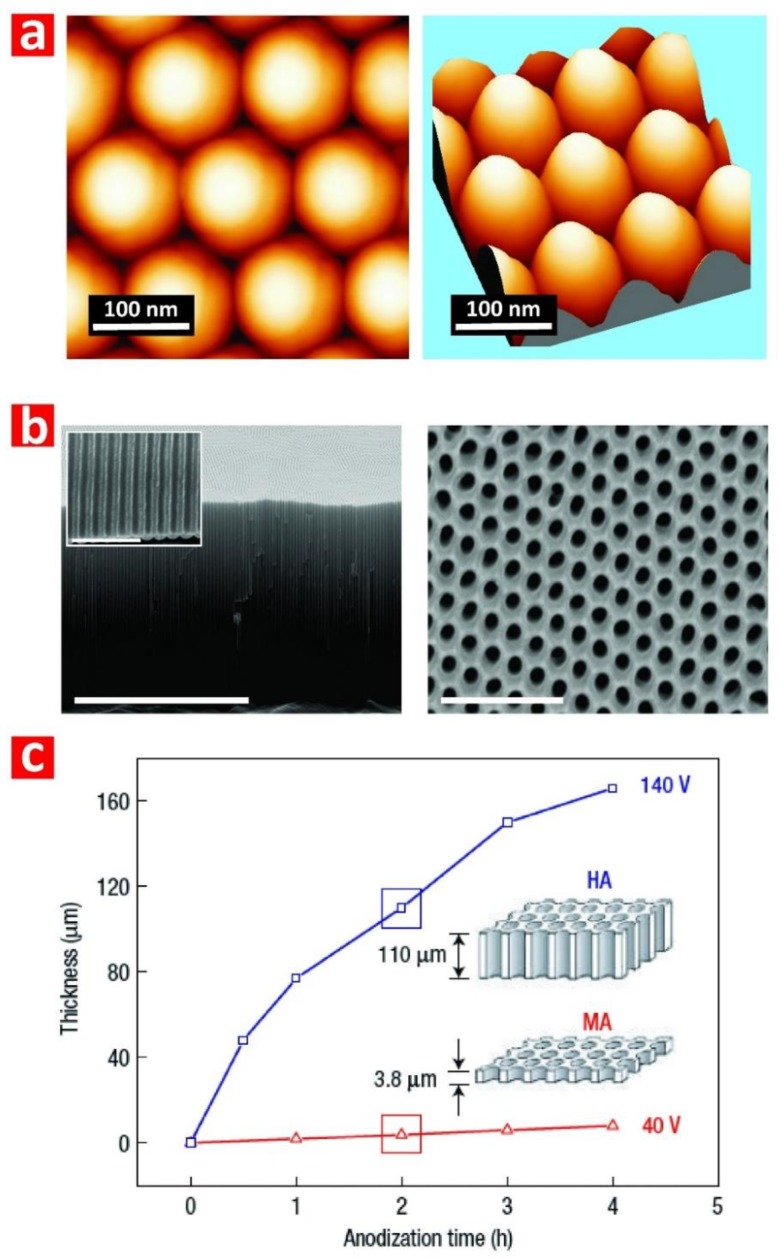
Self-organized nanoporous anodic alumina (NAA). (**a**) Atomic force microscopy images showing details of the hemispherical caps located at the bottom side of NAA produced by two-step anodization. (**b**) General cross-sectional FEG-SEM view of NAA featuring straight cylindrical nanopores from top to bottom (scale bar = 5 µm) with inset showing a magnified view of the oxide barrier layer (scale bar = 500 nm) and top FEG-SEM view of hexagonally arranged cylindrical nanopores in NAA (scale bar = 500 nm). (**c**) Film thickness as a function of anodization time at mild (MA at 40 V—red line) and hard anodization (HA at 140 V—blue line) with schematics showing the different thicknesses of anodic oxides after 2 h of anodization. Reproduced from [33], with copyright permission from Springer Nature, 2006.

The two-step anodization process is a top-down nanofabrication approach that consists of three stages: (i) formation of a sacrificial layer of NAA with randomly distributed nanopores at its top (i.e., first step); (ii) selective removal of the resulting NAA film by wet chemical etching; and (iii) re-anodization of the aluminum substrate (i.e., second step) (Figure 2b). During the first anodization step, nanopores grow randomly across the surface of the NAA film and self-organize as they grow due to a combination of mechanical stress between adjacent nanopores and electric field-assisted dissolution and growth of the oxide barrier layer at the electrolyte-oxide and oxide-metal interfaces, respectively (Figure 2c). This mechano-electrochemical self-ordering process patterns the surface of the underlying aluminum substrate, which is a negative replica of the bottom part of the sacrificial NAA layer featuring self-organized hemispherical caps (Figure 3a). During the second anodization step, nanopores grow at the center of each hemispherical void patterned on the aluminum substrate surface and propagate from top to bottom maintaining the self-ordered distribution. This simple approach enables the generation of highly ordered nanoporous structures (Figure 3b) without the need of sophisticated lithographic methods, opening new opportunities to generate a broad range of nanostructures in a fast, simple, and cost-competitive manner.

Anodization of aluminum is an electrochemical process in which two electrodes (i.e., anode = aluminum and cathode = platinum) are partially immersed in a mild acid electrolyte (Figure 2c). The growth of NAA is driven by the application of a voltage or current density between the two electrodes that leads to competing oxidation (i.e., formation of oxide at the metal–oxide interface) and dissolution (i.e., dissolution of oxide at the oxide–electrolyte interface) processes occurring simultaneously at the interfaces of the oxide barrier layer located at the nanopore bottom tips (Figure 2c). Anodization is an electric field-assisted electrochemical process in which the application of an external electric field drives the flow of ionic species (i.e., Al^3+^, O^2−^, OH^−^, and H^+^) involved in the formation and dissolution of oxide at the interfaces of the oxide barrier layer (i.e., metal/oxide and oxide/electrolyte interfaces) [28]. NAA structures can be produced by two anodization regimes: (i) mild anodization (MA), which is performed at low anodization voltage/current density and moderate acid electrolyte temperatures, and (ii) hard anodization (HA), which is characteristically carried out at high anodization voltage/current density and low acid electrolyte temperatures [33]. Whereas the characteristic growth rate of NAA under MA conditions is slow (i.e., 3–8 μm h^−1^), the growth of NAA films produced under HA regime is fast (i.e., 50–70 μm h^−1^) (Figure 3c). Furthermore, NAA produced under MA and HA conditions also differ in their geometric features and chemical composition (i.e., level of impurities). For example, whereas NAA-MA films produced in oxalic acid have a characteristic porosity of ~10%, the porosity of NAA-HA films is typically ~3%. Some excellent review articles covering different fabrication methods and characteristics of NAA are given in the literature [28,34,35].

### 2.2. Physical Properties of Nanoporous Anodic Alumina

**Figure 4:**
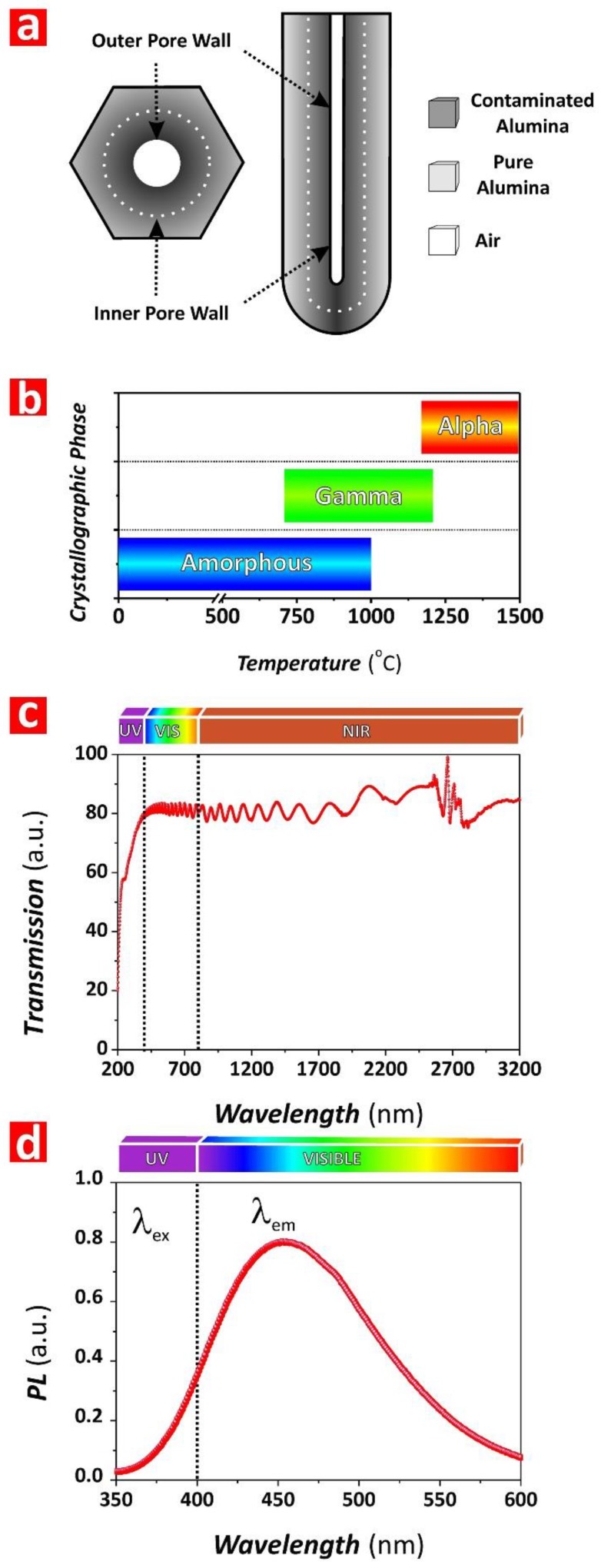
Physical properties of nanoporous anodic alumina (NAA). (**a**) Onion-like chemical structure of NAA with impurities distribution around the central nanopore. (**b**) Crystallographic phases of NAA as a function of temperature. (**c**) Representative UV-visible-near-infrared (NIR) transmission spectrum of NAA (note: NAA produced by two-step anodization under MA regime in oxalic acid electrolyte). (**d**) Representative photoluminescence spectrum of NAA (note: NAA produced by two-step anodization under MA regime in oxalic acid electrolyte).

NAA is a mechanically hard and brittle oxide that can withstand high pressures and temperatures. As-produced NAA has an onion-like chemical structure basically composed of Al_2_O_3_ with some water and impurities incorporated from the acid electrolyte during anodization (Figure 4a). NAA undergoes changes in its crystallographic phase when its structure is annealed (i.e., amorphous Al_2_O_3_ ~*T*_room_–1000 °C; gamma Al_2_O_3_ ~700–1200 °C; alpha Al_2_O_3_ ~1100–1500 °C), making it extremely resistant to acid or basic chemical etching (Figure 4b) [36]. With a refractive index of ~1.70 RIU, NAA has good transmittance (i.e., ~80–90%) in the UV-visible-NIR spectral region, although it significantly absorbs light in the UVC region (i.e., <280 nm) due to its intrinsic photoluminescence (PL) properties (Figure 4c). The origin of NAA’s PL relies on two types of PL centers: (i) F^+^ centers associated with ionized oxygen vacancies present in the structure of alumina and (ii) F centers attributable to impurities incorporated into the alumina structure from the acid electrolyte during anodization [37,38]. The PL of NAA is intrinsically dependent upon different fabrication parameters such as anodizing current density/voltage, type and concentration of acid electrolyte, crystallographic phase, and pore-widening treatment [39]. Typically, NAA features PL when it is excited at wavelengths <350 nm and its characteristic PL spectrum can be approximated to a Gaussian bell, the central wavelength and intensity of which can be tuned by the fabrication conditions (Figure 4d). Several studies have demonstrated that the characteristic PL spectrum of NAA films fabricated with certain geometric features (i.e., porosity and thickness) shows optical interference fringes generated by constructive reflection of internal light emission by Fabry–Pérot effect (Figure 4d) [39,40,41,42,43]. This intrinsic property of NAA can be readily used to develop optical sensing systems.

**Figure 5:**
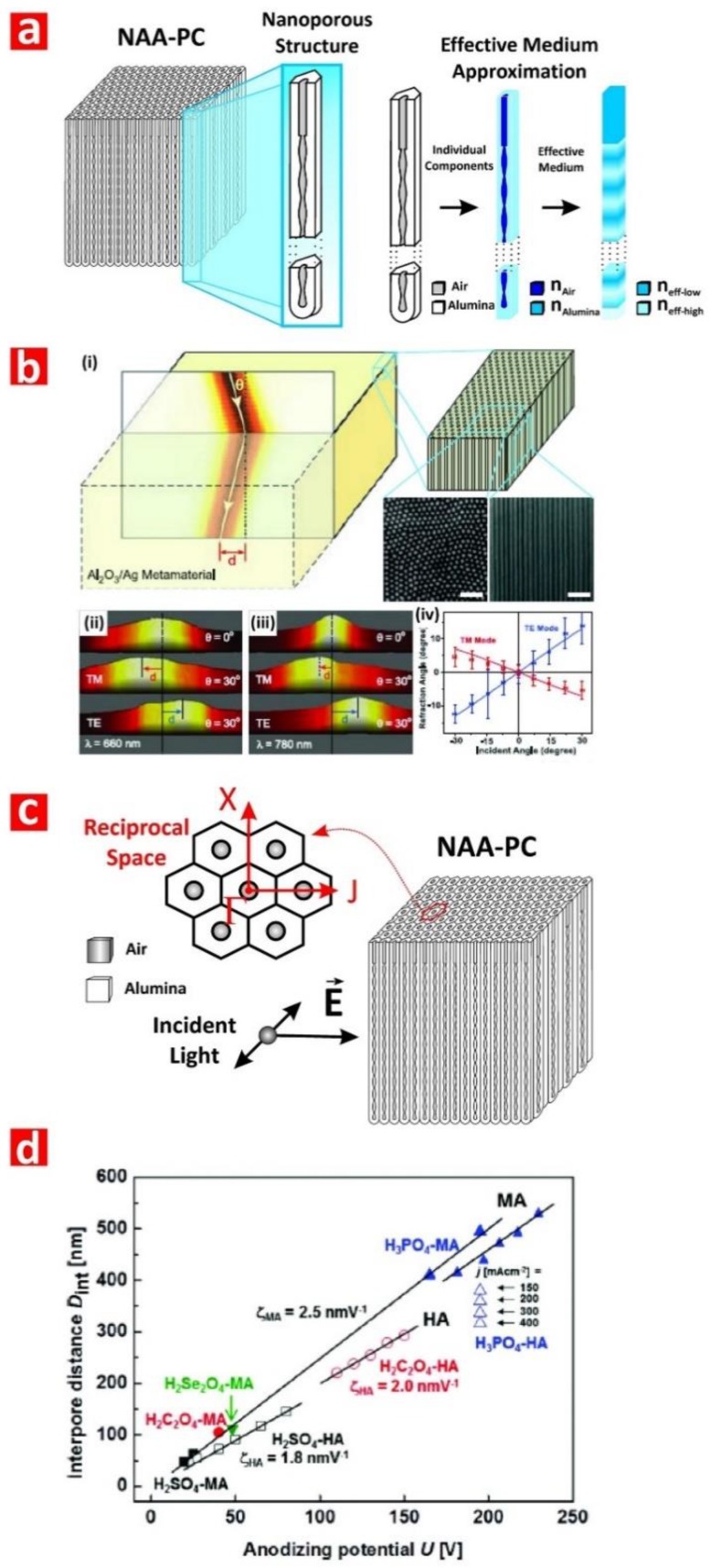
Intrinsic relationship between nanoporous geometry and optical properties in nanoporous anodic alumina photonic crystals (NAA-PCs). (**a**) Structural engineering of effective medium of NAA-PCs with details of the intrinsic relationship between nanoporous geometry and effective medium approximation. (**b**) NAA-PC as a metamaterial platform with negative refraction of light (reproduced from [45], with copyright permission from The American Association for the Advancement of Science, 2008). (**i**) Illustration showing negative refraction from air into composite NAA-PC filled with silver nanowires (**left**) and schemed showing the structure of the composite NAA-PC-Ag nanowires and SEM images of these photonic structures (scale bars = 500 nm); (**ii**) and (**iii**) beam intensities at the surface of the composite NAA-PC-Ag nanowires at 660 and 780 nm, respectively; (**iv**) dependence of refraction angles on incident angles and polarizations at 780 nm wavelength. (**c**) Schematic showing the structure of a 2D NAA-PC with incident light flowing perpendicularly to its nanopores. (**d**) Self-ordering regimes in MA (filled symbols) and HA (open symbols) in the most representative acid electrolytes used to produce NAA. Reproduced from [28], with copyright permission from American Chemical Society, 2014.

From an optical standpoint, the most attractive property of NAA is its versatile and highly controllable nanoporous structure, which can be readily utilized as an effective medium platform to develop unique PC structures to modulate and control the flow of electromagnetic waves with precision [29]. NAA is a binary composite matrix formed by air (i.e., 1 RIU) and alumina (i.e., ~1.70 RIU), in which the spatial distribution of these two components at the micro/nanoscale establishes the macroscopic optical properties of this nanomaterial (Figure 5a). The optical properties of NAA-PCs (i.e., effective refractive index and effective dielectric constant) can be estimated by averaging the properties of the individual constituents (i.e., air and alumina) as described by different effective medium approximation models, including Maxwell–Garnett, Bruggeman, Lorentz–Lorenz, Monecke, Drude, and Looyenga–Landau–Lifshitz. Selection of the most optimal effective medium model to correlate and describe the optical properties of the composite PC material is strongly dependent on the spatial distribution (i.e., PC structure—e.g., distributed Bragg reflector, gradient-index filter, etc.) and the intrinsic properties of the different components (i.e., refractive indices/dielectric constants) [44]. It is worth noting that the nanoporous structure of NAA also enables the generation of composite PCs by filling or coating its nanopores with other materials by means of different deposition techniques (e.g., electrodeposition, atomic layer deposition, infiltration, etc.). This approach provides multiple opportunities to create PC structures with extraordinary optical properties to attain a versatile control of light across the spectral regions (Figure 5b) [45].

Pioneering studies on NAA-PCs focused on analysis of light–matter interactions in NAA-PC platforms featuring hexagonally arranged straight cylindrical nanopores from top to bottom produced by a combination of nanoimprint lithography and one-step anodization or self-organized NAA-PCs produced by two-step anodization [46,47,48,49]. Light in these NAA-PCs is modulated when photons flow transversally across the composite matrix of alumina featuring cylindrical nanopores filled with air (Figure 5c). The seminal studies by Masuda and coworkers and Gösele and coworkers revealed that organized NAA-PCs feature a characteristic photonic stopband (PSB) in their optical spectrum, the position of which can be finely tuned by the interpore distance (i.e., lattice constant—distance between the center of adjacent nanopores) and porosity (i.e., pore diameter) of the NAA-PC platform. During the last decade, intensive research activity has aimed at extending the self-organization regime of NAA toward longer interpore distances (i.e., from 60 to 700 nm) by using different acid electrolytes (e.g., sulfuric, oxalic, selenic, malonic, phosphoric, phosphonic, tartaric, citric, phosphonoacetic, etidronic, etc.) [50,51,52,53,54,55,56,57,58,59,60,61,62,63,64] (Figure 5d). However, the versatility of these PCs to control light across the spectral regions is constrained by the range of available interpore distances. It is worth noting that the photonic band structure of NAA raising from its nanopore periodicity has yet to be utilized in optical sensing applications. Pulse-like anodization approaches have overcome this limitation by enabling the in-depth and multidimensional engineering of the NAA’s effective medium through structural engineering, paving the way for NAA-PC structures with controllable photonic features across the spectral regions.

### 2.3. Structural Engineering of Nanoporous Anodic Alumina

Pioneering studies by O’Sullivan and Wood established a direct relationship between the anodization voltage and the nanopore diameter in NAA, where the latter varies at a rate of ~1.29 nm V^−1^ with the former [65,66]. Therefore, pulse-like dynamic modifications of the anodization voltage during the growth of NAA could be used to induce nanopore modulations and engineer the structure of NAA. However, the oxide barrier layer located at the nanopore bottom tips of NAA is an electrical and ionic insulator that limits the direct translation of anodization voltage/current density modifications into nanopore diameter modulations. The thickness of the oxide barrier layer is directly proportional to the applied anodization voltage at an average rate of ~1 nm V^−1^ [67]. When the external electric field is suddenly modified during anodization, the flow of ionic species across the oxide barrier layer is altered, undergoing a recovery process that is strongly dependent upon the electric field change and the thickness of the oxide barrier layer [68,69,70].

In a series of pioneering works, Lee and coworkers developed pulse-like anodization approaches to engineer and modulate the porosity of NAA in a multidimensional fashion. The combination of anodizing voltage or current density pulses switched between MA and HA regimes was demonstrated as an effective approach to engineer the inner porosity of NAA with precision [71,72,73,74]. These strategies, based on the finding of different porosity levels between HA and MA (e.g., ~3 and ~10% in oxalic acid for HA and MA regimes, respectively), were devised by Lee and coworkers to controllably engineer the inner nanoporous structure of NAA with precision and versatility [33]. An optimal design of the anodization conditions such as the pulse anodization profile, acid electrolyte, and temperature enable the effective translation of electric field variations into nanopore diameter modulations, overcoming the limitations imposed by the oxide barrier layer while preventing nanopore branching [68,69,70]. These approaches inspired and boosted further fundamental and applied research to develop new anodization methods to engineering the nanoporous structure of NAA to generate PC structures.

While MA-HA pulse-like anodization approaches enable the precise modulation of the nanopore geometry in NAA, they do face some challenges due to the limited controllability of anodization under HA conditions. For instance, the growth rate of NAA under HA is extremely fast (i.e., 50–70 μm h^−1^) and it relies upon the nanopore length. Furthermore, the generation of Joule’s heat during HA requires the process to be performed at low acid electrolyte temperatures (i.e., ~0 °C). Alternative pulse-like anodization strategies performed under MA conditions provide a controllable means of tuning the optical properties of NAA-PCs. Although the slow oxide growth rate under MA conditions (i.e., 3–8 μm h^−1^) is a limiting factor, it enables the precise engineering of the NAA’s effective medium to attain a better control over the features of the photonic stopband (PSB) of NAA-PCs across the spectral regions.

**Figure 6:**
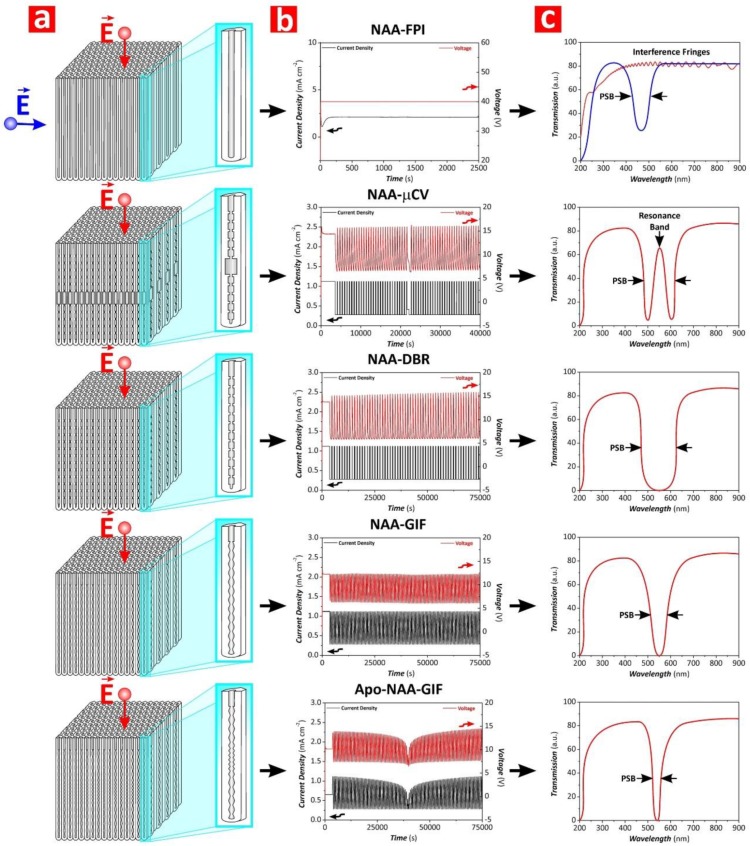
Characteristic nanoporous geometry, anodization profile, and optical properties of representative nanoporous anodic alumina photonic crystals (NAA-PCs) (from top to bottom: NAA-FPI—NAA Fabry–Pérot interferometer; NAA-μCV—NAA optical microcavity; NAA-DBR—NAA distributed Bragg reflector; NAA-GIF—NAA gradient-index filter; Apo-NAA-GIF—apodized NAA gradient-index filter). (**a**) Schematic showing details of the nanoporous structure of NAA-PCs where $\vec{E}$ indicates the direction of the electromagnetic field. (**b**) Representative experimental anodization profiles used to produce NAA-PCs. (**c**) Illustration of optical transmission spectra showing the characteristic photonic features of NAA-PCs.

In recent years, numerous studies have realized and developed different NAA-PC architectures, the most representative examples of which are Fabry–Perót interferometers (NAA-FPIs), optical microcavities (NAA-μCVs), and 3D NAA-PCs. In addition to these, different architectures of NAA-based distributed Bragg reflectors (DBRs) have been realized, including NAA-DBRs—this term will henceforth refer to NAA-DBRs produced by stepwise pulse anodization, gradient-index filters (NAA-GIFs), apodized DBRs and GIFs (Apo-NAA-DBRs and Apo-NAA-GIFs), bandpass filters (NAA-BPFs), and linear variable bandpass filters (NAA-LVBPFs). Figure 6 shows schematic illustrations of the structure of these NAA-based PC structures along with examples of anodization profiles used to produce these NAA-PCs and their representative optical spectra depicting the characteristic features of the PSB as a function of the NAA-PC architecture. Some excellent review articles have recently described the fundamental concepts and realization of NAA-PCs [29,34]. This review article provides a fresh and comprehensive collation of the most recent developments and applications of NAA-PCs in chemo- and biosensing applications, including surface chemistry modifications.

NAA-DBRs can be produced by stepwise pulse anodization (STPA) approach performed under different conditions (i.e., MA or MA-HA pulses) [75,76]. The structure of these NAA-PCs is characterized by a stepwise modulation of porosity, where the STPA profile is translated into nanopore diameter modulations that correspond to high and low levels of anodizing voltage or current density. The transmission spectra of these NAA-PCs show a characteristically broad PSB, the position and features of which can be readily tuned by different anodization parameters. NAA-GIFs feature a sinusoidal modulation of porosity and are fabricated by sinusoidal pulse anodization (SPA), a process in which the anodization voltage or current density is pulsed between high and low values in a sinusoidal fashion [77].

Transmission spectra of NAA-GIFs are characterized by a narrow, well-defined, and spectrally tunable PSB, which is a result of a smooth modulation of effective refractive index driven by the SPA profile. Apo-NAA-DBRs and Apo-NAA-GIFs are produced by apodizing STPA and SPA anodization profiles, respectively [78,79,80,81]. The characteristic PSB of these NAA-PCs is similar to that of their nonapodized counterparts but with much narrower width due to the apodization of their effective medium. Several studies have demonstrated the successful application of different apodization functions to STPA and SPA profiles to engineer the photonic features of NAA-DBRs and NAA-GIFs [78,79,80,81]. NAA-μCVs are a class of NAA-PCs that confine light to small volumes by resonant recirculation of electromagnetic waves [82,83,84]. Typically, the structure of NAA-μCVs is composed of a physical cavity layer featuring straight cylindrical nanopores, which is sandwiched between two highly reflective mirrors (e.g., NAA-DBRs, NAA-GIFs, etc.). However, other NAA-μCV architectures have been identified [85]. Light reflection by the mirrors forming the structure of NAA-μCVs is maximum at those wavelengths where light interferes in these PCs in a constructive fashion, which is denoted by the characteristic PSB. The introduction of a cavity layer between the mirrors forming the structure of NAA-μCVs creates destructive interferences that lead to the generation of a resonance band within the characteristic PSB. The characteristics of the cavity layer and the mirrors establish the conditions where light confinement is at a maximum. NAA-FPIs are composed of straight cylindrical nanopores that feature a homogeneous distribution of effective refractive index in depth. The optical spectrum of these NAA-PCs has a characteristic PSB when light flows transversally through the NAA-FPIs’ structure, which is established by the interpore distance (i.e., lattice constant) and porosity (i.e., nanopore diameter) [46,47,48,49]. NAA-BPFs are PC structures that allow the transmission of a specific portion of the light spectrum in a selective manner while impeding the pass of light of all other wavelengths [79,86]. NAA-BPFs can be classified into three categories according to the range of allowed wavelengths: (i) longpass filters, which allow the transmission of light of long wavelengths, (ii) shortpass filters, which allow the pass of light of short wavelengths, and (iii) bandpass filters, which allow the transmission of a band of wavelengths while blocking the pass of light of shorter and longer wavelengths. However, other types of NAA-BPFs with complex transmission bands (i.e., several transmission bands located at different sections of the UV-visible-NIR spectrum) can be fabricated. NAA-LVBPFs have a PSB with variable central wavelength, the position of which is shifted across the surface of the filter in a linear fashion. This effect is achieved by a selective etching of the nanoporous structure of NAA-LVBPFs to engineer their effective medium in the perpendicular direction to the nanopores’ growth [87].

## 3. Surface Modification of Nanoporous Anodic Alumina Photonic Crystals

**Figure 7:**
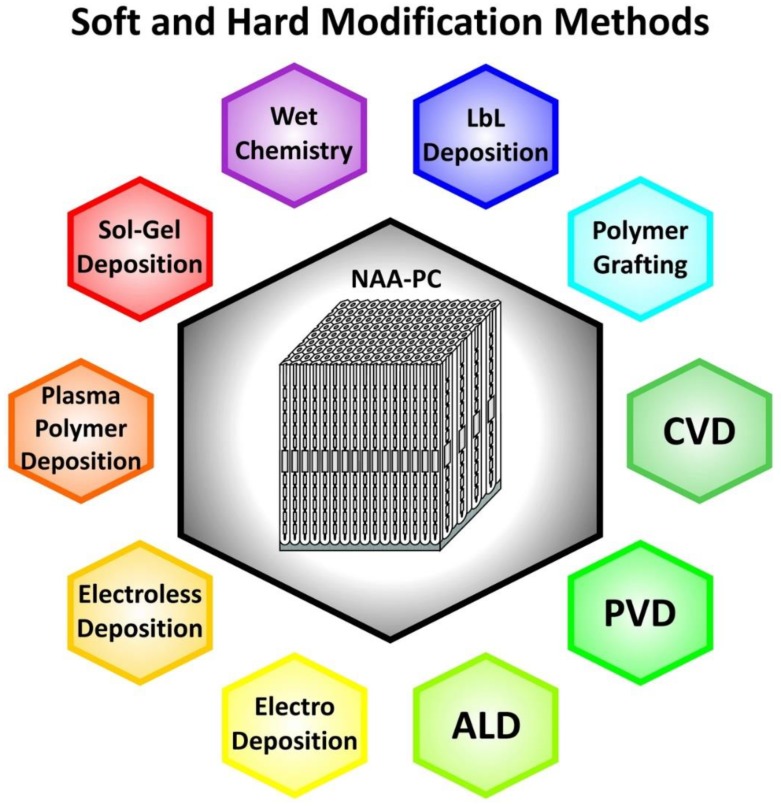
Soft and hard deposition methods used to modify the physical and chemical properties of NAA-PCs.

NAA-PCs have an onion-like layered structure consisting of two main layers: an inner layer away from the central nanopore at the aluminum-alumina interface and an outer layer close to the central nanopores located between the inner layer and the alumina-electrolyte interface (Figure 3a) [88]. The major constituent of the inner layer is pure alumina (Al_2_O_3_), whereas the outer layer contains anionic contaminants incorporated from the acid electrolyte during anodization such as sulfate, oxalate, and phosphate [29]. Some studies reveal the presence of more than two chemical layers in NAA-PCs. Yamamoto et al. identified three layers in NAA, as suggested by the PL spectrum of NAA after discontinuous pore-widening steps [89]. Santos et al. identified four layers in the chemical structure of NAA with an increasing concentration of impurities toward the layer near the central nanopore by real-time monitoring of pore-widening of NAA using reflectometric interference spectroscopy [90]. During anodization, heterolytic dissociation of water molecules occurs at the oxide-electrolyte interface, leading to the generation of hydroxyl groups on inner surface of NAA-PCs [91]. The presence of hydroxyl groups on the inner surface of NAA-PCs allows surface modifications by binding different functional molecules with desirable functionalities for specific sensing applications [92]. A number of different surface modification methodologies is still available and these can be categorized into soft and hard techniques (Figure 7) [93].

### 3.1. Soft Chemical Modification Techniques

#### 3.1.1. Wet Chemistry

The formation of self-assembled monolayers (SAMs) provides a method to functionalize NAA-PCs with organic functional molecules (i.e., aliphatic and aromatic) via anchoring groups such as thiols, disulfides, amines, silanes, and acids. The formation of SAMs is induced by spontaneous strong chemisorption or physisorption of the functional moiety of organic molecules to a solid substrate, which leads to the orderly arrangement of organic films with controlled thickness. When a substrate is immersed in a diluted solution of absorbate for a given time, the organic molecules attach and arrange themselves in an energetically stable form at the solid–liquid interface, imparting the substrate functional entities for different applications [93]. Despite some limitations such as potential oxidation, electric field-induced, and thermal desorption, SAMs generated by wet chemical technique provide many advantageous properties. These include a tight and dense packing configuration of functional groups to attain chemical selectively toward target analytes, chemical stability after immobilization of target molecules, anti-biofouling properties, and flexibility of functional moiety and molecular size. For instance, SAMs of alkanoic acid can be generated in NAA-PCs to alter their surface chemistry [93]. Tao et al. and others studied and analyzed the formation of SAMs based on n-alkanoic acids with chain lengths from C4 to C20 and C24 in NAA [94,95,96]. SAMs of alkanoic acids with different side functional groups and fluorinated alkanoic acid can also be generated onto the surface of NAA by anchoring carboxylate groups to its surface while providing other functionalities on the outer edge of the monolayer [97,98]. SAMs of alkanoic acids with dual functional groups allow the secondary functional group to be available for further functionalization with other molecules (i.e., immunoglobulin G and octylamine) [96,98]. Organophosphates form highly stable and densely-packed SAMs due to their phosphonic acid head group (i.e., phosphorus tetrahedrally bonded to carbon, oxygen atoms, and two hydroxyl groups), which forms a covalent bond with hydroxyl groups present on the inner surface of NAA [99]. Organophosphate-modified NAA can be used to detect transition metals for ion exchange, selective sorption, and catalysis [100]. Organophosphate SAMs can also be used as linkers to organic matrices such as pyrrole-containing molecules and graphene oxide in multicomponent systems [91]. Various phosphonic acids such as alkylphosphonic acid [101], fluorinated phosphonic acid [102], and ester-containing phosphonic acid [103] have been used to modify NAA platforms by forming organophosphorus SAMs. SAMs based on bifunctional phosphonic acids and allow further surface reactions of the terminal group with other functional molecules such as polymers, biomolecules, and small molecules [97,104,105].

Other popular organic molecules used to form SAMs on NAA platforms are organosilanes, which are a group of chemical compounds derived from silanes containing one or more organic groups. The silanol group of organosilanes attaches covalently to the hydroxyl groups present on the surface of NAA [88]. These functional molecules can be immobilized onto NAA platforms by wet chemical technique and chemical vapor deposition (CVD), by which evaporated organosilane molecules deposit and self-organize on NAA [106]. A variety of silanes has been explored to modify the properties of as-produced NAA for different purposes, including tuning its surface wettability, improving its biocompatibility, or altering the effective pore geometry for chemical selectivity in various applications such as molecular transport and separation, immunoisolation, hydrophylization, and sensing. SAMs of silanes with hydrophobic terminal groups increase the hydrophobicity of NAA [107], SAMs of perfluoroalkylchorosilanes reduce the effective pore diameter of NAA for molecular transport [108], poly(ethylene glycol)silane SAMs improve the biocompatibility of NAA for immunoisolation [109], and SAMs of mercaptosilanes have been used for sensing applications [110,111,112,113]. Similar to SAMs of alkanoic and phosphonic acids, SAMs of organosilanes can act as chemical linkers for further immobilization of biomolecules [114,115,116], polymers [116], and nanoparticles [117]. Furthermore, organosilanes containing different terminal functionalities (e.g., amine, thiol, carboxyl, etc.) can be deposited onto the inner surface of a single NAA platform in a differential fashion to provide desirable gradient functionalities for specific applications such as and multiplexed sensing [118,119]. Organosulfur is also used to create SAMs on NAA by wet chemical route. The formation of organosulfur-based SAMs requires the pretreatment of NAA platform, which involves the deposition of a thin layer of gold by electroless, electrodeposition, or sputtering technique [91]. The sulfhydryl or disulfide moiety of organosulfur molecules has strong affinity toward gold, leading to relatively strong bonds that form stable SAMs [120,121]. The resulting organosulfur-based SAMs can be used for various applications such as support layer for lipid bilayer formation [122] as well as further functionalization for chemo- and biosensing [123]. Alkene and alkynes are able to form SAMs on NAA by thermally-induced wet chemical technique [97,124]. Alkene- and alkyne-based SAMs modify the hydrophobicity of NAA platforms and enable further chemical functionalization for sensing applications [97,125]. SAMs of alkynes are more stable than their alkene-based equivalents. The instability of alkene-based SAMs is attributed to the different molecular species formed after binding to hydroxyl groups present on the surface of NAA [97]. Alkene SAMs undergo a rapid decrement of carbon content and contact angle upon exposure to phosphate buffer saline solution, which denotes their faster degradation rate as compared to their alkene counterparts. Comparative studies have evaluated the stability and quality of SAMs formed on the surface of NAA platforms using different functional molecules. Bhairamadgi et al. tailored the surface of NAA with SAMs of phosphonic acid and alkyne molecules and assessed their hydrolytic and thermal stability. Phosphonate monolayers on NAA were relatively stable when immersed in acidic and neutral solutions but showed poorer stability under alkaline conditions. The thermal stability of phosphonate-based SAMs on NAA was found to be exceptional, with only a 10% loss of monolayer due to thermally induced desorption at temperatures as high as 562 °C [124]. This was associated with the strong binding of phosphonic acids to hydroxyl groups on NAA via monodentated, bidentated, or tridentated structures [126]. On the other hand, alkyne monolayers on NAA showed weaker hydrolytic and thermal stabilities as compared to phosphonate-based SAMs. These SAMs showed a decreasing contact angle and carbon-to-aluminum ratio with temperature as well as a lower temperature at 10% monolayer desorption (284 °C) [124]. Similar to phosphonates, alkynes are capable of forming bidentate structures with surface hydroxyl groups present on the surface of NAA [125]. However, the nature of the chemical bond (Al–O–C) is more prone to dissociation as compared to that of phosphonates (Al–O–P) [97]. Debrassi et al. evaluated the stability of chemically modified NAA platforms with SAMs of phosphonate, carboxylate, alkene, alkyne, and silane molecules. The order of stability based on the amount of carbon on SAMs-modified NAA immersed in phosphate buffer saline solution was found to be phosphonate > silane > alkyne > alkene > alkanoic acid [97]. The stronger stability of phosphonate-based SAMs on NAA was further proven over a range of pHs (pH 4–8) and temperatures (25–80 °C) [127]. The strengths and weaknesses of different types of SAMs are summarized in Table 1.

Wet chemical techniques can also be used to build substrate-supported biomimetic lipid membranes. The adsorption of lipid vesicle dispersions to NAA platforms yields lipid bilayers that mimic cell membranes, which can be used as a model to investigate molecular processes occurring at membrane level, concomitant membrane structural and fluidity changes, as well as a support for biological sensing events between membrane and proteins [128]. The formation of lipid bilayers usually involves fusion of lipid vesicles on either bare or functionalized NAA platforms [122]. Typically, bare NAA platforms are directly exposed to lipid vesicle dispersions by either immersion or wetting by droplets, leading to the formation of lipid nanotube arrays [129,130]. On the other hand, NAA platforms can be pretreated as a preparation for subsequent lipid functionalization. In this process, NAA is coated with a gold layer that is used to capture thiol-containing lipids and to link molecules with sulfhydryl moiety for subsequent lipid binding [122,131]. Another approach to deposit lipid molecules on NAA is by utilizing a support layer of silane, sometimes coupled with cross-linkers (e.g., polymers and streptavidin-biotin), where lipid molecules bind to the reactive surface functional groups electrostatically or covalently [128,132,133]. Wet chemical technique also offers a simple route for the immobilization of charged molecules onto the inner surface of NAA via electrostatic interactions [134]. Protonated hydroxyl groups and exposed cationic aluminum ions present on the surface of NAA can interact with anions when NAA is incubated in a solution of functionalizing molecules. It is important to ensure that the incubation time is long enough for effective electrostatic immobilization. An additional hydroxylation step can be performed in order to create more hydroxyl groups on the surface of NAA. In these electrostatic interactions, proteins that are negatively charged at pH higher than their isoelectric point (e.g., protein A, urea, and bovine serum albumin) can be immobilized onto positively-charged NAA. These functional layers can be used to develop a broad range of optical biosensor [135,136,137].

#### 3.1.2. Layer by Layer Deposition

Layer by layer deposition (LbL) is a versatile and simple surface modification technique to create functional and multilayered thin films on solid or nanoporous platforms. The thin film is created by alternating dipping stages of a substrate into solutions containing functional molecular species with an intermediate rinsing step in between for removal of weakly-adsorbed molecules and minimization of cross-contamination. Although dip coating is an efficient and automatable approach, this technique is time consuming due to the multiple steps at low speed required to achieve homogeneous coatings. Alternative methods such as alternated spray deposition and spin coating can address this limitation, although these techniques have other intrinsic drawbacks such as waste of depositing solutions and limited area of deposition. LbL of a wide variety of functional molecules can be applied to various surfaces such as planar substrates, colloids, nanoparticles, and porous materials [138]. The underlying principle of LbL assembly is based on electrostatic interactions between positively and negatively charged molecules. However, other approaches such as LbL of layers by chemical interactions such as hydrogen bonding, metal-ligand coordination chemistry, hydrophobic interactions, and biological recognition are possible [139]. These approaches allow the deposition of LbL films beyond conventional and functional polyelectrolytes, including biomolecules (i.e., DNA, nucleic acids, proteins, and viruses), nanoparticles, organic dyes, dendrimers, and inorganic molecules [140,141]. The thickness of thin films created by LbL can be controlled with nanometric precision, with multiple interactions between the layers of the resulting film providing enhanced stability [141]. The LbL method has been used to tailor the surface chemistry of NAA in terms of transport properties and permeability as well as further functionalization [108]. Polyelectrolytes such as poly(stryrene sulfonate) and poly(ally amine hydrochloride) are commonly used for deposition of LbL films on NAA [142,143,144]. Multilayered polyelectrolyte films can also be generated on NAA using templated polyelectrolytes and functional polyelectrolytes such as dendrimers [145,146]. This approach enables further functionalization with nanoparticles [147,148], drug molecules [149], antibodies [149], DNA [150], and ions [151]. DNA strands and proteins (i.e., glucose oxidase, hemoglobin, and cytochrome C) can be assembled layer by layer inside the nanopores of NAA by hybridization and by protein immobilization agents based on covalent bonding and electrostatic adsorption [152,153]. Typically LbL assembly on NAA platforms may require pretreatment with silanes to generate a positively-charged surface [142,146].

#### 3.1.3. Polymer Grafting

The inner surface of NAA can be modified by polymers using two approaches: (i) the “grafting-to” method involving the anchoring of polymer brushes to a solid interface and (ii) the “grafting-from” method in which the initiator molecules lead to the grow polymer chains on the inner surface of NAA [154]. Although the former method is relatively simple, it only provides limited grafting densities of polymer brushes and film thickness [155]. On the other hand, the “grafting-from” approach offers a better control over the polymeric film in terms of structure, thickness and density. Among different surface-initiated polymerization techniques (i.e., reversible addition-fragmentation chain-transfer polymerization, ring opening metathesis polymerization, and iniferter polymerization), atom-transfer radical polymerization (ATRP) have been extensively explored for the generation of polymer brushes. Unlike LbL films, polymers grown from surfaces display a more extended and less cross-linked configuration, which is more suitable to develop swollen films that are able to bind a higher number of biomacromolecules [156]. Polymerization of NAA often requires a prefunctionalization step such as silanization, gold coating for adsorption of thiol-containing initiators, generation of hydroxyl groups for subsequent attachment of initiators or grafting of polymers such as poly(N-isopropylacrylaminde, poly(methacrylic acid), and poly(oligo (ethylene glycol) methyl ether methacrylate) [157,158]. Modification of NAA by polymer grafting enables the tuning of the surface properties of NAA such as permeability, wettability, and chemical selectivity [159,160] as well as other features, including immobilization of molecules such as proteins, nanoparticles, and steroids [161,162,163]. Surface-initiated polymerization techniques can be combined to create films based on different polymers within NAA [164]. This technique is also compatible with the LbL method to construct composite polymer films inside the nanopores of NAA [165].

### 3.2. Hard Chemical Modification Techniques

#### 3.2.1. Chemical Vapor Deposition

The surface properties of NAA can be modified by chemical vapor deposition (CVD), technique by which the inner surface of NAA is coated by a thin film of the deposited material through dissociation and chemical reaction between a gaseous reactant and the surface of NAA with the aid of heat, light, or plasma. CVD enables the generation of uniform coatings with controllable structure and good conformal coverage. This functionalization method is versatile and allows the deposition of many different chemical precursors such as metals, carbides, oxides, sulfides, silanes, and semiconductors. However, the deposition of multicomponent films by CVD is limited by due to different vaporization rates of precursors [166]. CVD is commonly used to grow nanotubes and nanowires based on semiconductor materials, carbons, and polymers [167,168,169,170]. CVD is also employed for the formation of silane monolayers onto NAA as an alternative modification to conventional wet chemistry approaches due to its high reproducibility, reduced chance of particle contamination, and silanol oligomerization [171]. Organosilane modification of NAA by CVD allows further functionalization such as molecule immobilization and polymerization [116,170,172]. These CVD modification methods have been used to develop drug delivery systems, sensors, and electronic devices [173,174].

#### 3.2.2. Physical Vapor Deposition

Physical vapor deposition (PVD) is a hard modification technique used to deposit thin films of elements, alloys, and compounds on NAA platforms [175]. PVD involves the physical transformation of analytes to gaseous state by thermal evaporation or impact process followed by their deposition onto the surface of NAA [176]. PVD can be classified into different categories such as electron beam evaporation, thermal evaporation, sputter deposition, pulsed laser deposition, and molecular beam epitaxy [28]. Properties such as conductivity, reflectivity, and chemical stability and, also, for further chemical modifications, enhancing interactions with various chemical and biological species for optical sensing and molecular separation applications can be improved by modification of the structure of NAA platforms with different coatings produced by PVD [92]. Gold, platinum, nickel, indium-tin oxide, manganese, silver, semiconductor oxides, and mixtures of these are often deposited onto NAA platforms by electron beam evaporation, sputtering or shadow evaporation to produce nanostructured material platforms for sensing, imaging, photocatalysis, and photovoltaics due to their desirable plasmonic and optoelectronic properties [121,177,178]. These functional coatings enable further chemical modifications of NAA with a wide range of molecules such as thiolates, lipids, and polyelectrolytes [120,179,180].

#### 3.2.3. Atomic Layer Deposition

Atomic layer deposition (ALD) is another hard modification method used to deposit thin and conformal films that provides precise control over the thickness and composition of the film at atomic scale. ALD is based on a self-limiting and layer-by-layer approach that enables the deposition of a broad range materials including oxides, nitrides, phosphates, sulfides, and metals. ALD involves alternating saturate surface reactions of pulsing and purging precursors [181]. The main advantage of ALD is its ability to form homogenous monolayers over large areas and high aspect ratio nanoporous substrates due to its three-dimensional nature [182]. Furthermore, monolayers formed by ALD are versatile in their composition and thickness. However, ALD has some intrinsic drawbacks such as its slow deposition rates, potential cross-contamination of thin films by residual precursors, and high costs for certain materials [183,184]. Despite these limitations, ALD provides high reproducibility and flexibility and it has been extensively used to modify NAA platforms for various applications [129,185]. Conformal single and doubled layered structures based on a broad range of materials such as silicon dioxide, titanium oxide, sulfides, palladium, and platinum deposited onto NAA platforms by ALD have been explored not only for surface modifications, but also to fabricate nanostructures by template synthesis such as nanotubes and nanowires [186,187]. ALD-modified NAA has been used to develop sensors, energy materials, and membranes [188,189,190]. Polymers such as polyimide can also be deposited onto NAA membranes via ALD for separation applications [191]. Furthermore, ALD coatings deposited onto NAA platforms provide a means for further functionalization, opening up new opportunities for advanced sensing applications [192].

#### 3.2.4. Electrochemical Deposition

Electrochemical deposition has also been used to modify NAA with metal and alloys, where the coating is produced by current or voltage driven electrochemical reduction reactions within NAA platforms [193]. Metals are typically deposited inside the nanoporous network of NAA from electrolytes, which serves as cathode during this process. Despite its limitations, such as low deposition rate and single use of host template, electrodeposition is an attractive approach to surface-modify NAA platforms and prepare nanostructures such as nanotubes or nanowires since it is cost-effective, simple, and it can be performed with simple laboratory equipment at room temperature [194,195]. Nanostructures based on different metals (i.e., copper [196], nickel [197], and antimony [198]) have been successfully synthesized in NAA platforms by electrodeposition, enabling the precise tailoring of the properties of these materials for specific applications such as sensing and catalysis [28,199]. Metal oxides and alloys can also be electrodeposited in NAA platforms to produce nanowires and nanotubes [200,201]. The versatility of this technique is further demonstrated by its ability to synthesize nanoparticles, nanocomposites, and multilayered nanostructures in NAA platforms [198,202,203]. These structures can be further functionalized with thiols, alkanoic acids, and proteins for various purposes such as drug delivery and sensing [204,205]. However, the composition of multilayered nanostructures is not limited to metals since other composite structures such as conducting polymers (e.g., polypyrrol) can be successfully integrated with gold to produce multisegmented nanowires [206].

#### 3.2.5. Electroless Deposition

Electroless deposition can be used to deposit metals and alloys onto NAA platforms without the need of external current as it is based on a purely chemical process (i.e., displacement and autocatalytic deposition) based on oxidation-reduction reactions in aqueous or nonaqueous solutions [207]. Electroless deposition requires reducing agents such as formaldehyde and polydroxyl alcohols or surface activation agents. The advantage of electroless deposition is that this method can be applied to coat the surface of nonelectronically conductive platforms such as NAA [91]. The coatings formed by electroless deposition are uniform, continuous, and replicate the geometrical characteristics of nanoporous templates. NAA has been long devised as a template for electroless deposition of metals in the form of nanotubes and nanoparticles including cobalt, nickel and zinc [208,209,210]. The formation of gold coatings in NAA platforms by electroless deposition provides plasmonic and catalytic properties and these initial gold layers can be used as a precursor for further functionalization [210,211,212,213].

#### 3.2.6. Plasma Polymer Deposition

Plasma polymerization is also used to functionalize the surface of NAA platforms with polymer films. This process utilizes organic and inorganic precursors to grow polymeric thin films with the aid of plasma discharge to catalyze these chemical reactions [214]. Polymeric films formed by plasma polymerization are highly cross-linked, randomly branched, and lack regular repeating units, making them mechanically, chemically, and thermally strong. Compared to other polymer deposition methods, plasma polymer deposition offers a high degree of versatility and control as the chemical composition and thickness of the resulting films can be manipulated easily by means of the deposition parameters and the nature of the precursors. Furthermore, this technique uses a small amount of reactants for polymeric film deposition at low temperature, making it cost-effective [214]. Nonetheless, polymer plasma deposition cannot achieve sufficient penetration depth to coat the inner surface of nanoporous materials such as NAA, being limited to surface-based applications [106]. Modification of NAA platforms with plasma polymer deposition enables the tuning of the hydrophobicity of the surface by polymerizing fluorocarbon monomers using argon or oxygen plasma [215]. Plasma polymer deposition also changes the chemical and geometrical properties of NAA as platform material in drug delivery systems, where drug release from NAA can be controlled and extended over longer periods of time [216,217]. This technique also allows the deposition of functional monomers to create chemically reactive polymeric surfaces that can be further functionalized for various applications including molecular separation, optical sensing, and drug delivery [218].

#### 3.2.7. Sol-Gel Chemistry Deposition

The sol-gel method can be used to alter the surface chemistry and geometrical characteristics of NAA platforms and to fabricate various nanostructures by template synthesis. This process is divided into three steps: (i) hydrolysis and partial condensation of a precursor solution by immersion, dipping, or spin coating, (ii) gel formation by polycondensation, and (iii) solvent evaporation and gel drying [219,220]. A variety of materials can be obtained via the sol-gel method as it allows direct preparation of sol-gel composite films with controllable stoichiometry and homogeneity at low processing temperature. Sol-gel derived films possess high specific surface area and a surface with rich chemistry that allows ease of functionalization [221]. On the other hand, the sol-gel process has some disadvantages such as limited thickness and mechanical weakness of the resulting film [222]. Careful consideration and handling of precursors must be done since some precursors are too unstable to form sol-gel films [219]. However, sol-gel chemistry modification is an extensively used method to tune the surface characteristics of NAA platforms and to synthesize nanocomposite materials. The flexibility of sol-gel chemistry to modify NAA platforms is demonstrated by the wide range of materials that can be deposited by this technique such as metal oxides (i.e., titanium dioxides [223], silica [224], and zinc oxide [224]) as well as mixtures of inorganics [225,226]. The surface modification of NAA by sol-gel can be further expanded via the immobilization of molecules onto the sol-gel derived films. Sol-gel modified NAA platforms can be endowed with biofunctionalities by immobilization of biomolecules, gold nanoparticles, and drug-bearing polymers. These platforms can be applied in immunoassays, biosensing, and drug delivery applications [227,228]. Furthermore, sol-gel derived functional coatings synthesized within NAA platforms (i.e., titanium and tin oxides) provide composite photocatalyst materials for environmental remediation and green energy generation applications [223,229].

## 4. Nanoporous Anodic Alumina Photonic Crystals as Optical Sensing Platforms

NAA-PCs show much potential as advanced and versatile optical sensing platforms due to their unique physical, chemical, and optical properties. To engineer the effective medium and surface chemistry of NAA-PCs enhances the sensing performance and capabilities of NAA-based optical sensing system in terms of selectivity, sensitivity, and specificity. The geometric features of nanopores in NAA-PCs can be precisely engineered by different anodization approaches to selectively filter molecules by size-exclusion and increase the available binding functional sites due to the high specific surface area to volume ratio of these nanoporous PCs. Furthermore, the surface chemistry of NAA-PCs can be modified with different functional molecules through well-established protocols to achieve chemical selectivity toward target molecules and analytes [34]. NAA-PCs are active optical platforms that confine, guide, reflect, emit, and transmit incident light, generating stable optical signals for chemo- and biosensing applications based on different spectral shifts upon exposure to analyte molecules such as reflectivity, photoluminescence, transmittance, waveguiding, absorbance, or color changes. Different forms of NAA-PCs (e.g., distributed Bragg reflectors, grading-index filters, optical microcavities, Fabry–Pérot interferometers, etc.) can be integrated with various optical techniques such as reflectometric interference spectroscopy (RIfS), reflection and transmission spectroscopy, and photoluminescence spectroscopy (PL) (Table 2) [29,87,230]. This section summarizes the most representative examples of chemo- and biosensing systems combining NAA-PC structures with optical techniques.

### 4.1. Nanoporous Anodic Alumina Distributed Bragg Reflectors (NAA-DBRs)

NAA-DBRs are typically fabricated by stepwise pulse anodization (STPA) process, in which the anodizing current density or voltage is pulsed in a stepwise fashion by means of MA or MA-HA pulses. The nanoporous structure of NAA-DBRs features a stepwise modulation of porosity, with stacks of NAA layers of alternating porosity that follow the high and low levels of anodization voltage or current density applied during the STPA. NAA-DBRs have a broad PSB, which shifts its position when the effective medium of these PCs is modified upon exposure to analytes of interest. This property can be used to develop a series of optical chemo- and biosensing systems.

**Figure 8:**
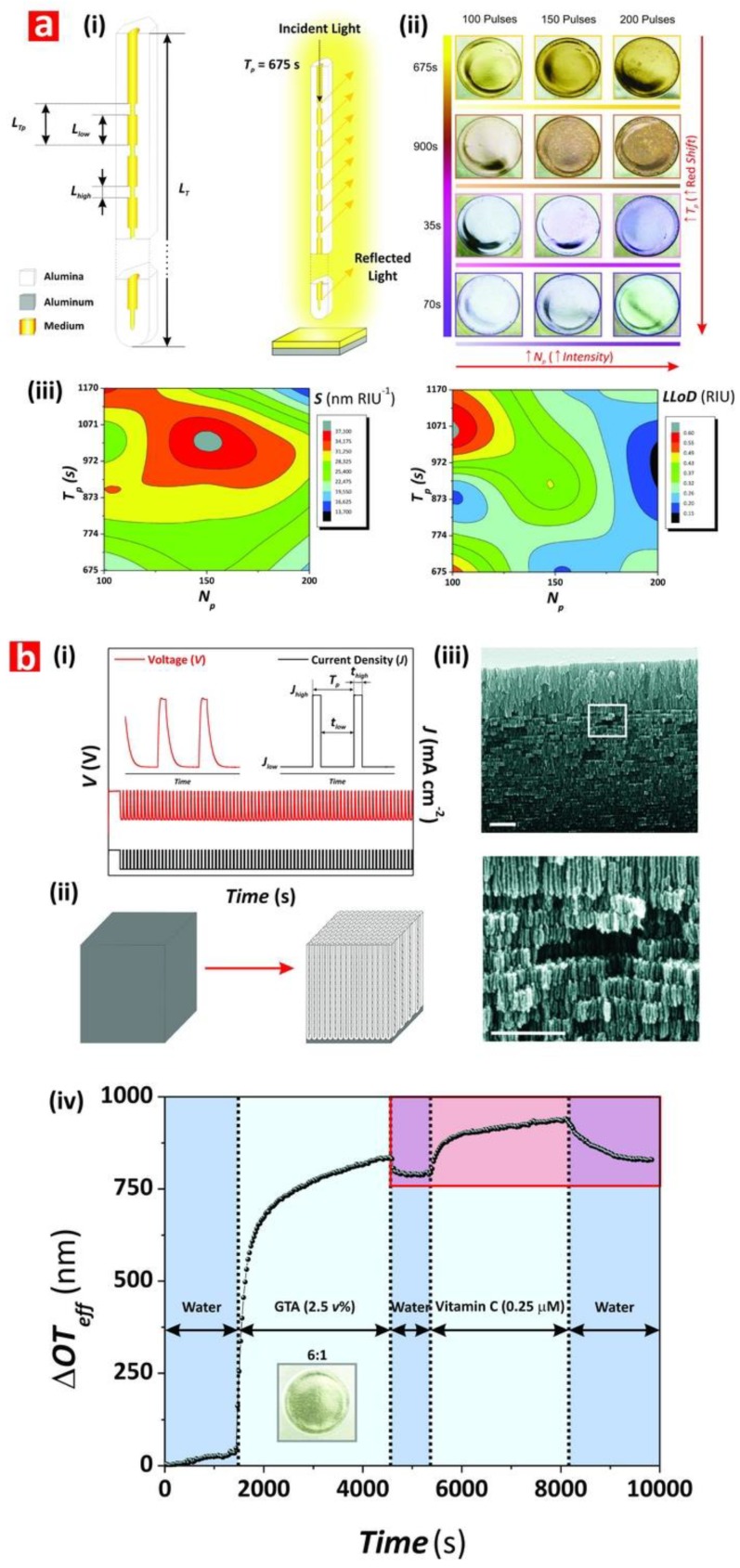
Examples of applicability of NAA-PCs in chemo- and biosensing applications. (**a**) (**i**) Schematic showing details of the structure of Nanoporous anodic alumina-distributed Bragg reflectors (NAA-DBRs) produced by STPA, (**ii**) digital pictures showing the interferometric colors displayed by these NAA-PCs, and (**iii**) sensitivity and low limit of detection as a function of the fabrication parameters. Reproduced from [231], with copyright permission from American Chemical Society, 2015. (**b**) (**i**) STPA profile used to produce NAA-DBRs, (**ii**) schematic showing the fabrication of NAA-DBRs by STPA, (**iii**) SEM images of NAA-DBRs, and (**iv**) real-time effective optical thickness change for each sensing stage of vitamin C. Reproduced from [232], with copyright permission from American Chemical Society, 2015.

Chen et al. fabricated NAA-based DBRs by galvanostatic pulse anodization under mild conditions in sulfuric acid electrolyte. The structure and optical properties of these NAA-DBRs where tuned across the spectral regions by a systematic modification of anodization period (*T_p_*) and the number of pulses (*N_p_*) (Figure 8a) [231]. The optical properties of these NAA-DBRs were assessed by reflectometric interference spectroscopy (RIfS) and compared in terms of sensitivity (*S*), low limit of detection (*LLoD*), and linearity (*R*^2^). The effective medium of these NAA-DBRs was infiltrated with solutions of different reactive index (i.e., D-glucose, ethanol, and isopropanol), leading to quantifiable changes in the effective optical thickness (∆*OT_eff_*) of these NAA-PCs by RIfS. NAA-DBRs produced with *T_p_* = 1035 s and *N_p_* = 150 pulses showed the highest sensitivity toward changes in the effective medium, with *S* = 37,931 nm RIU^−1^, *LLoD* = 0.352 RIU, and *R*^2^ = 0.9876. These NAA-PCs were then utilized as sensing platforms for selectively detection of gold (III) ions (*Au*^3+^) in combination with RIfS. The surface chemistry of NAA-DBRs was modified with 3-(mercaptopropyl)-trimethoxysilane (MPTMS) by CVD to generate thiol functionalities onto the inner surface of these PCs. A linear correlation between ∆*OT_eff_* and the concentration of *Au*^3+^ ([*Au*^3+^]) was used to establish the characteristic sensing parameters of these platforms, which were *S* = 22.2 nm µM^−1^, *LLoD* = 0.16 µM, and *R*^2^ = 0.9983. In another study by Chen et al., the authors developed an optical sensing system combining NAA-DBRs with RIfS for the detection of vitamin C (Figure 8b) [232]. A set of NAA-DBRs with different optical and geometric properties was fabricated by a systematic manipulation of *T_p_*, anodization temperature (*T_an_*), and time ratio of high and low current density values (*R_t_*) in the STPA profile. The nanopores of these NAA-DBRs were infiltrated with a mixture of ethanol and isopropanol in different ratios to assess the effective medium sensitivity in terms of *S*, *LLoD*, and *R*^2^ by RIfS. The highest sensitivity (*S* = 27,553 nm RIU^−1^) was achieved by NAA-DBRs fabricated with *T_p_* = 675 s, *T_an_* = 3 °C, and *R_t_* = 6:1. A set of these NAA-DBRs was then functionalized with (3-aminopropyl)-trimethoxysilane (APTES) to achieve chemical specificity toward vitamin C molecules.

Different concentrations of vitamin C solutions were used to characterize the sensing performance of APTES-functionalized NAA-DBRs through real-time measurements of ∆*OT_eff_* using RIfS. The performance of this sensing system for vitamin C was *S* = 227 nm µM^−1^, *LLoD* = 20 nM, and *R*^2^ = 0.9985. These NAA-DBRs displayed vivid interferometric colors, which were demonstrated to be precisely tunable (i.e., brown, gold, pink, purple, and green) by modifying different fabrication parameters (i.e., anodization temperature, anodization period, ratio between time at high, and low current density) [232]. Color changes in these NAA-PCs were used as a sensing principle to develop platforms for colorimetric sensing. NAA-DBR films were broken down into nanoporous microparticles by sonification and infiltrated with media of different refractive index such as air and isopropanol. Color changes associated with effective medium changes were then quantified in terms of RGB values. These NAA-DBR nanoporous microparticles are suitable to develop microsensors and self-reporting nanocarriers.

Law et al. applied apodization to the STPA profile used to produce Apo-NAA-DBRs under current density control conditions [233]. The effective medium of these NAA-DBRs was systematically engineered by modifying *T_p_*, from 1100 to 1700 s, and the pore-widening time (*t_pw_*), from 0 to 6 min. The sensitivity of apodized and nonapodized NAA-DBRs was assessed by infiltrating their nanopores with mediums of different refractive index (i.e., air, ethanol, and water). Spectral shifts in the position of the characteristic PSB of these NAA-DBRs upon infiltration with different mediums were monitored in real-time by RIfS and used to assess the optical properties of these NAA-PCs. The linear correlation between shifts in the position of PSB and the refractive index of the medium filling the nanopores demonstrated that apodized NAA-DBRs were ~16% more sensitive (*S* = 392 nm RIU^−1^) than their nonapodized counterparts (*S* = 339 nm RIU^−1^). The obtained results also revealed that a longer *T_p_* (1700 s) and a moderate *t_pw_* (4 min) enhance the sensitivity of NAA-DBRs for optical sensing applications. Kumeria et al. fabricated NAA-DBRs produced with porosity modulated in a pseudosinusoidal fashion. These NAA-PCs were used as sensing platforms in combination with RIfS to develop an optical system for the detection of mercury ions (Hg^2+^) under specific adsorption conditions [111]. Functionalization of the inner surface of these NAA-DBRs with thiol-silane molecules provided NAA-DBRs with chemical selectivity toward Hg^2+^. Changes in the position of the characteristic reflection band of NAA-DBRs upon exposure to different concentrations of Hg^2+^ were monitored in real-time by RIfS. This sensing system achieved a sensitivity of 0.0115% μM^−1^, a low limit of detection of 4.20 μM, and *R*^2^ = 0.994. Guo et al. demonstrated the use of NAA-based Bragg stacks as chemical sensors for in situ monitoring of organics with varied refractive index [230]. NAA-DBRs were used as colorimetric sensing platforms, where color in NAA-DBRs changed from blue to green upon exposure to ethanol. To further characterize the sensitivity of these NAA-PCs, the transmission spectra of NAA-DBRs exposed to a series of alkanes (i.e., n-hexane, n-octane, and n-decane) and alcohols (i.e., anhydrous ethanol, 2-propanol, 1-butanol, and 1-hexanol) were analyzed.

**Figure 9:**
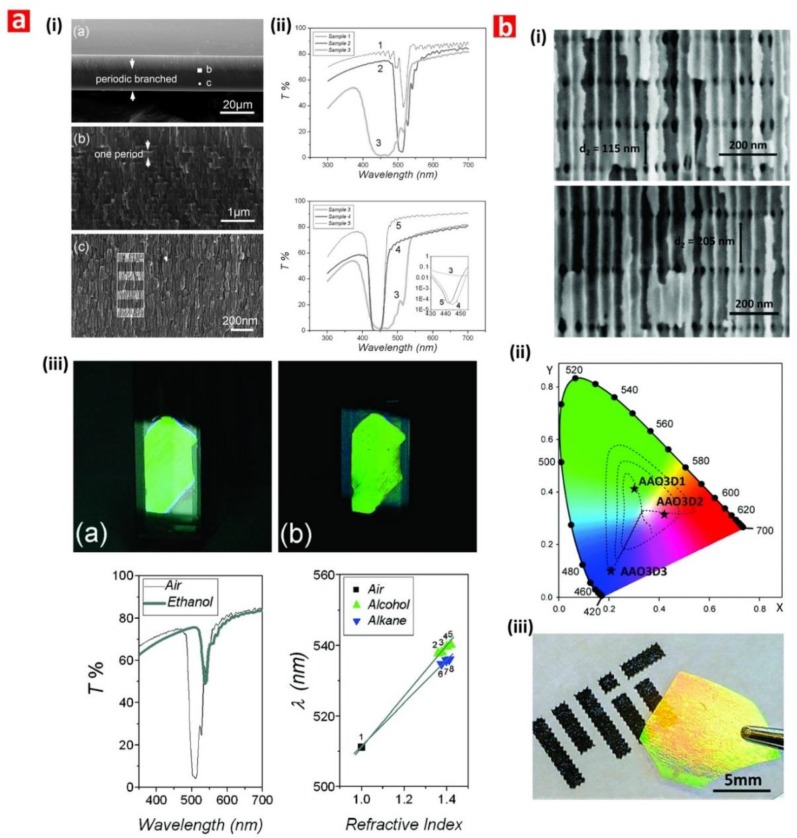
Examples of applicability of NAA-PCs in chemo- and biosensing applications. (**a**) (**i**) SEM images of NAA-DBRs, (**ii**) transmission spectra of NAA-DBRs as a function of the fabrication conditions, and (**iii**) interferometric colors, transmission spectra, and position of the PSB for different media filling, Reproduced from [234], with copyright permission from American Chemical Society, 2008. (**b**) (**i**) SEM images of NAA-DBRs, (**ii**) position within the CIE color space, and (**iii**) digital images showing the interferometric color of NAA-DBRs at different fabrication conditions. Reproduced from [235], with copyright permission from American Chemical Society, 2018.

Spectral shifts in the characteristic photonic stopband in the transmission spectrum of NAA-DBRs were used to establish a relationship with the refractive index of media filling the nanopores. The sensitivity of these NAA-PCs was 71.4 and 61.9 nm RIU ^−1^ for alcohols and alkanes, respectively. The use of NAA-DBRs as visual sensing tool was further explored by Chen et al. for chemically selective detection of mercury ions (Hg^2+^) [110]. A palette of structurally colored NAA-DBRs was produced as a function of *T_p_* (from 675 to 1170 s) and *T_an_* (from −1 to 3 °C). These NAA-DBRs red-shifted their characteristic interferometric color upon infiltration of their nanoporous matrix with ethanol. An analysis of color changes and ∆*OT_eff_* measured before and after infiltration by RIfS established that NAA-DBRs produced at *T_p_* = 1035 s and *T_an_* = −1 °C displayed the sharpest change in color and the highest effective optical thickness change. A set of these NAA-DBRs was then further developed as a chemically selective visual sensing platform to detect mercury ions. These NAA-DBRs underwent quantifiable color changes during the different sensing stages, including functionalization with thiol-terminated functional groups and exposure to Hg^2+^. RGB values of NAA-DBRs as a function of the concentration of Hg^2+^ ([Hg^2+^]) revealed a linear correlation between the intensity of the blue and green channels in the RGB color and [Hg^2+^] from 10 to 100 mM. An analysis of the sensing performance established a sensitivity and a low limit of detection of 0.81 a.u. µM^−1^ and 1.25 µM for the blue channel and of 29.4 a.u. µM^−1^ and 37.3 µM for the green channel, respectively. Guo et al. developed NAA-DBR platforms to detect organic molecules through shifts in the characteristic photonic stopband in the transmission spectrum of these NAA-PCs (Figure 9a) [234]. NAA-DBRs were produced by a modified two-step anodization process and exposed to a series of alcohols (i.e., anhydrous ethanol, 2-propanol, 1-butanol, and 1-hexanol) and alkanes (i.e., *n*-hexane, *n*-octane, and *n*-decane). Spectral shifts in the PSB of these NAA-PCs upon exposure to analytical solutions demonstrated that NAA-DBRs have higher sensitivity toward alcohols and alkanes of longer chains due to the higher refractive index of these analyte molecules. Ruiz-Clavijo et al. developed a colorimetric sensor based on NAA-DBRs with periodic composition of high-porosity and low-porosity layers (Figure 9b) [235]. The colorimetric sensing properties of these NAA-DBRs were assessed in terms of interferometric color shift upon infiltration of nanopores with water and background substrate. The color variations in NAA-DBRs were quantified by the CIE color space chromaticity diagram.

### 4.2. Nanoporous Anodic Alumina Gradient-Index Filters (NAA-GIFs)

NAA-GIFs show a sinusoidally periodic variation of effective refractive index that is typically produced by the sinusoidal pulse anodization process (SPA). This smooth variation of effective refractive index gives rise to a much narrower and well-defined PSB, which is more sensitive toward changes in the effective refractive index of the NAA-PC platform [236,237].

Kumeria et al. fabricated NAA-GIFs by a modified two-step anodization process, in which the second step consisted of a pseudosinusoidal voltage profile controlled by total charge under potentiostatic conditions [238]. The sensitivity of NAA-GIFs toward changes in their effective refractive index were assessed by RIfS. NAA-GIFs were exposed to aqueous analytical solutions of D-glucose with concentration ranging from 0.01 to 1.00 M, equivalent to a modification of refractive index from 1.333 to 1.363 RIU. Shifts in the position of the characteristic PSB produced by changes in effective refractive index upon infiltration were used to establish a linear correlation between spectral shift and refractive index of media filling the nanopores. These NAA-based PCs achieved a sensitivity for glucose of 4.93 nm M^−1^ (i.e., 164 nm RIU^−1^), with a *LLoD* of 0.01 M, and a *R*^2^ of 0.998. These NAA-GIFs also displayed vivid interferometric colors that could be precisely tuned across the visible spectral region by the anodization parameters and pore-widening time. The infiltration of the nanoporous network of NAA-GIFs with acetone led to dynamic color changes from intense green to intense red due to alteration of the effective medium of these NAA-PCs. This demonstrated the capability of NAA-GIFs as colorimetric platforms to develop visual sensing tools.

**Figure 10:**
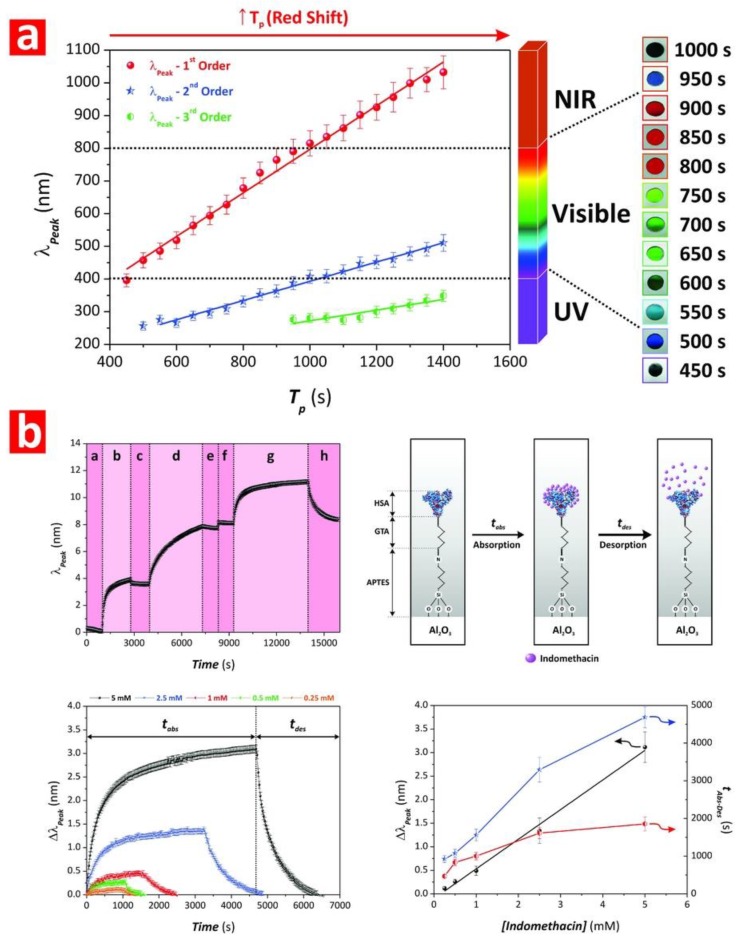
Examples of the applicability of NAA-PCs in chemo- and biosensing applications. (**a**) Dependence of position of PSB and anodization period of NAA-GIFs produced by SPA with digital pictures showing the interferometric colors displayed by these NAA-PCs. (**b**) Sensing process and description of sensing stages used to assess the affinity between drugs and human serum albumin using NAA-GIFs as sensing platforms. Reproduced from [77], with copyright permission from Royal Society of Chemistry, 2016.

Santos et al. developed an optical sensing system combining protein-modified NAA-GIFs and RIfS to assess the pharmacokinetic profile of drugs (Figure 10a,b) [77]. NAA-GIFs were fabricated by sinusoidal pulse anodization under galvanostatic conditions using sulfuric acid as an electrolyte. These NAA-PCs were chemically functionalized with silane molecules and human serum albumin (HSA) molecules were immobilized onto the surface of APTES-modified NAA-GIFs via glutaraldehyde activation. HSA-modified NAA-GIFs were exposed to indomethacin, a model drug, and the binding events occurring between immobilized HSA molecules and free indomethacin molecules were monitored in real-time by RIfS. The position of characteristic PSB of HSA-modified NAA-GIFs was found to red-shift when these PC platforms were exposed to different concentrations of indomethacin. A linear correlation between spectral shift and concentration of drug was used to establish the sensing performance of this system, with results: *S* = 0.63 nm mM^−1^, *LLoD* = 0.065 mM, and *R*^2^ = 0.9935. This study demonstrated the applicability of the proposed system for pharmacokinetic assessment of drug–protein interactions. In an extension of this study, Nemati et al. assessed systematically the binding affinity between a set of drug molecules and HSA using optimized NAA-GIFs as sensing platforms and RIfS [171]. Spectral shifts in the characteristic PSB and changes in the effective optical thickness of HSA-functionalized NAA-GIFs upon exposure to various drug molecules (i.e., sulfadymethoxine, coumarin, warfarin, indomethacin, and salicylic acid) were assessed as a function of the concentration of model drug. The affinity between HSA-modified NAA-GIFs and drug molecules, defined by the slope of the linear fitting between the sensing parameter and the concentration of drug, was found to be dependent on the sensing parameter used (i.e., PSB shift or effective optical thickness change).

**Figure 11:**
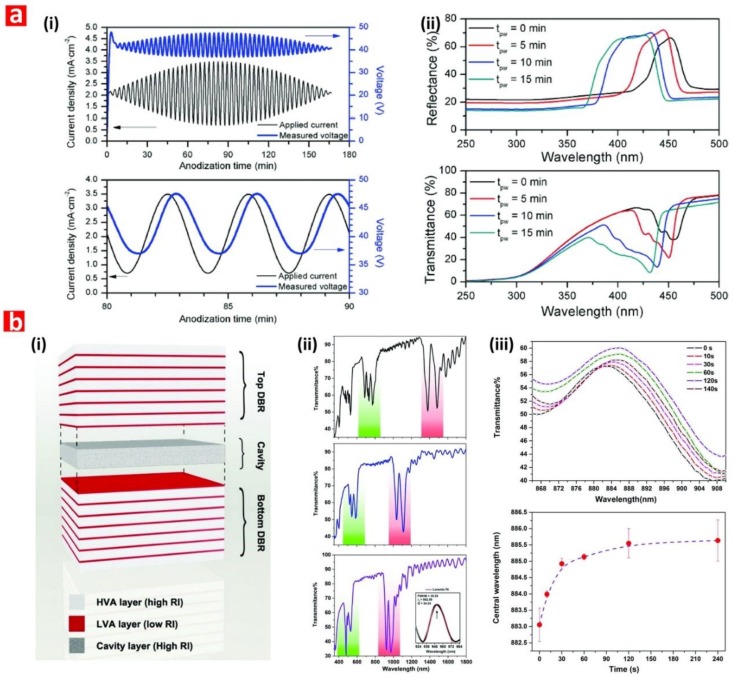
Examples of applicability of NAA-PCs in chemo- and biosensing applications. (**a**) (**i**) SPA profile used to produce NAA-GIFs and (**ii**) reflection and transmission spectra of NAA-GIFs as a function of the pore-widening time. Reproduced with permission from authors of a previous paper [81]. Copyright Springer, 2014. (**b**) (**i**) Schematic showing the structure of NAA-μCVs with two NAA-DBR mirrors and a layer of constant porosity in between, (**ii**) transmission spectrum of NAA-μCVs showing how the resonance band blue-shifts with the pore-widening time, and (**iii**) detection of humidity level based on red shifts in the resonance band of NAA-μCVs. Reproduced from [83], with copyright permission from American Chemical Society, 2015.

Kumeria et al. combined NAA-GIFs with RIfS to develop an optical sensing system to detect Hg^2+^ in aqueous solutions [112]. The sensing principle of this system relies on red shifts in the position of characteristic PSB of NAA-GIFs produced by the selective binding of Hg^2+^ to the thiol-functionalized surface of NAA-GIFs. The system’s performance was assessed for a range of concentrations from 1 to 750 μM. A linear fitting between the spectral shift and [Hg^2+^] established an *S* of 0.072 nm μM^−1^, a *LLoD* of 1 μM, a linearity of 0.992, and a linear working range from 1 to 100 μM. The chemical selectivity of this system was demonstrated through exposure of thiol-modified NAA-GIFs to analytical solutions containing interfering metal ions (e.g., Cu^2+^, Pb^2+^, and Fe^3+^) and aqueous matrices of different complexity such as ultrapure, tap, and river water. The obtained results verified the high selectivity of the sensing system toward Hg^2+^ without further chemical treatments, despite the presence of interfering elements. Marcias et al. fabricated NAA-GIFs using an apodized sinusoidal current density profile and assessed the sensing capabilities of these NAA-PCs upon infiltration of their nanoporous structure with media of different refractive index (Figure 11a) [81]. Spectral shifts in the characteristic PSB of NAA-GIFs after infiltration with air, ethanol and deionized water were determined by reflection spectroscopy. The sensing system was able to detect small changes in refractive index, with a sensitivity of 48.8 nm RIU^−1^.

### 4.3. Nanoporous Anodic Alumina Optical Microcavities (NAA-μCVs)

NAA-μCVs are a class of NAA-PCs that confine electromagnetic waves to microvolumes by resonant recirculation of light. The nanoporous structure of NAA-μCVs is typically composed of two highly reflective mirrors (e.g., NAA-DBRs, NAA-GIFs, etc.) between which a physical cavity layer featuring straight cylindrical nanopores is sandwiched. However, other forms of NAA-μCV architecture have been demonstrated.

Wang et al. engineered two types of NAA-μCV architectures by stepwise pulse anodization in oxalic acid electrolyte (Figure 11b) [83]. These PCs were used as platforms to develop a humidity sensor. The resonance band within the characteristic PSB in the transmission spectra of NAA-μCVs underwent red shifts when the sensing platforms were exposed to water vapor at different time intervals. The condensation of water molecules within the nanoporous structure of NAA-μCVs modified the effective medium of these NAA-PCs, leading to a red shift of the resonance band position of 2.58 nm. This study established the foundation for the use of NAA-μCVs in gas sensing applications. Yan et al. fabricated NAA-μCVs with a defect layer created by a constant voltage step in between a periodic sawtooth-like pulse voltage profile [84]. These NAA-PCs featured narrow resonance bands that were used as sensing parameter when the nanoporous network of NAA-μCVs was filled with liquids of different refractive index such as water, ethyl alcohol, and ethylene glycol. Shifts in the resonance band position upon infiltration were evaluated as a function of the refractive index of infiltrating medium. This sensing system was demonstrated to be capable of identifying analytes based on shifts in the resonance band position, with a sensitivity of 424.4 nm RIU^−1^. Lee et al. engineered NAA-μCVs by tuning the electrolyte temperature during a stepwise pulse anodization process [82]. NAA-μCVs were immersed in a series of polar (i.e., water, anhydrous ethanol, and isopropyl alcohol) and nonpolar (i.e., *n*-hexane, cyclohexane, and trichloroethylene) analytical solutions and red shifts in the position of the characteristic resonance band were used to establish the sensing performance of the system. This refractometric sensor was found to have a sensitivity of 441 nm RIU^−1^. The use of this sensing system as a colorimetric tool, based on structural color changes upon infiltration, was also demonstrated. NAA-μCVs were infiltrated with analytes of different refractive index such as air, water, isopropyl alcohol, cyclohexane, and trichloroethylene, which triggered dynamic colorimetric responses. Color changes were analyzed in terms of lightness and chromaticity in the CIELab 19,130 tristimulus color space. NAA-μCVs colorimetric sensors were able to detect refractive index differences of ~0.01 RIU, with a perceptual color change over the whole visible range. An et al. produced NAA-μCVs by a periodic pulse anodization approach with an effective voltage compensating strategy [239]. NAA-μCVs were chemically modified with rhodamine B by adsorption to form rhodamine B-NAA-μCVs composite sensing platforms. NAA-μCVs enhanced the photoluminescence intensity of the functional molecules absorbed onto the inner surface of NAA-μCVs. Although no sensing application was demonstrated, this system could potentially be used to develop PL-based sensors.

### 4.4. Nanoporous Anodic Alumina Fabry–Pérot Interferometers (NAA-FPIs)

NAA-FPIs feature straight cylindrical nanopores from top to bottom with a homogeneous distribution of effective refractive index. NAA-FPIs interfere constructively with electromagnetic waves, generating a characteristic interference pattern with distinctive fringes, which are a result of the Fabry–Pérot effect. Alterations of the effective medium of NAA-FPIs leads to shifts in their characteristic interference pattern, which can be used as sensing principle to create optical systems with broad applicability.

Kumeria et al. demonstrated the use of NAA-FPIs for optical sensing under both nonspecific and specific adsorption conditions [111]. NAA-FPIs were fabricated via two-step anodization in oxalic acid electrolyte. These NAA-PCs were exposed to different analytical solutions of glucose and their RIfS spectrum was analyzed to establish the effective optical thickness changes (∆*OT_eff_*) as a function of the refractive index of the glucose solution. The nonspecific infiltration of the effective medium of NAA-FPIs achieved an *S* = 18.42% RIU^−1^, a *LLoD* of 0.084 RIU, and a *R*^2^ of 0.998. NAA-FPIs were further assessed as chemically selective platforms for the detection of Hg^2+^. The performance of this sensing system for selective Hg^2+^ detection was *S* = 0.0009% μM^−1^, *LLoD* = 22.82 RIU, and *R*^2^ = 0.854.

Law et al. developed a sensing system combining NAA-FPIs and RIfS to monitor gold–thiol interaction [123]. NAA-FPIs were functionalized with a range of thiol-containing with different molecular features and backbone sizes. The sensing performance of the system was evaluated using analyte solutions of gold ions, which have high affinity toward thiol functional groups. Shifts in the effective optical thickness of NAA-FPIs upon exposure to gold ions were used as sensing principle to establish the sensing characteristics of the system. The highest sensitivity value, 5.6 nm μM^−1^, was achieved by NAA-FPIs featuring a dual functionalization of the inner and top surface with 6-amino-1-hexanethiol and 1,6-hexanedithiol, respectively. Rajeev et al. combined NAA-FPIs with RIfS to develop a biosensor for chronic wound care, using tumor necrosis factor-alpha (TNF-α) in buffer and simulated wound fluid as a biomarker [240]. The inner surface of NAA-FPIs was chemically modified with anti-TNF-α antibodies via silanization to achieve selectivity toward TNF-α. The sensitivity of this sensing system for TNF-α in buffer, assessed through ∆*OT_eff_* from varying TNF-α concentration, was determined to be 22.384 nm (ng mL^−1^)^−1^, with a *LLoD* of 0.13 μg mL^−1^. The selectivity and specificity of this biosensor to TNF-α was also verified in simulated wound fluid containing other interfering molecules.

The combination of NAA-FPIs with RIfS as a biosensing system to detect circulating tumor cells (CTC) was demonstrated by Kumeria et al. [241]. NAA-FPIs functionalized with biotinylated anti-EpCAM antibody through several surface modification steps have been used to detect Human pancreatic cancer cells (PANC-1) in whole blood and buffer. The different sensing stages were characterized in terms of ∆*OT_eff_* and CTC concentration by RIfS. The developed sensor was capable of selective detection of cancer cells with a concentration range from 1000 to 100,000 cells mL^−1^, with a *LLoD* < 1000 cells mL^−1^. The sensing capabilities of NAA-FPIs combined with RIfS for gas sensing were assessed by Kumeria et al. [242]. The adsorption of hydrogen sulfide gas (H_2_S) on gold-coated NAA-FPIs induced a concentration-dependence shift in the effective optical thickness of these films due to modification of the effective medium of these PC platforms. Real-time oral malodor monitoring was performed using the proposed device for a concentration range from 0.2 to 0.4 μg L^−1^. Casanova et al. monitored gas adsorption and liquid desorption of organic vapors in NAA-FPIs using optical interferometry to demonstrate the potential of NAA-FPIs combined with RIfS as gas sensor [243]. The interaction of toluene and isopropanol with the nanopores were translated into ∆*OT_eff_*. The sensing performance was found to be dependent on the partial pressure of the gas. This system was able to discern between different phase transitions such as monolayer–multilayer adsorption and capillary condensation. Santos et al. used a combination of NAA-FPIs and RIfS to monitor nonspecific and specific adsorption of glucose and L-cysteine, respectively [43]. A linear correlation between ∆*OT_eff_* and the concentration of analytes adsorbed in NAA-FPIs established a sensitivity of 0.007% mM^−1^ with a *LLoD* of 100 mM under nonspecific adsorption conditions and a sensitivity of 0.026% mM^−1^ and *LLoD* of 5 mM under specific adsorption conditions.

**Figure 12:**
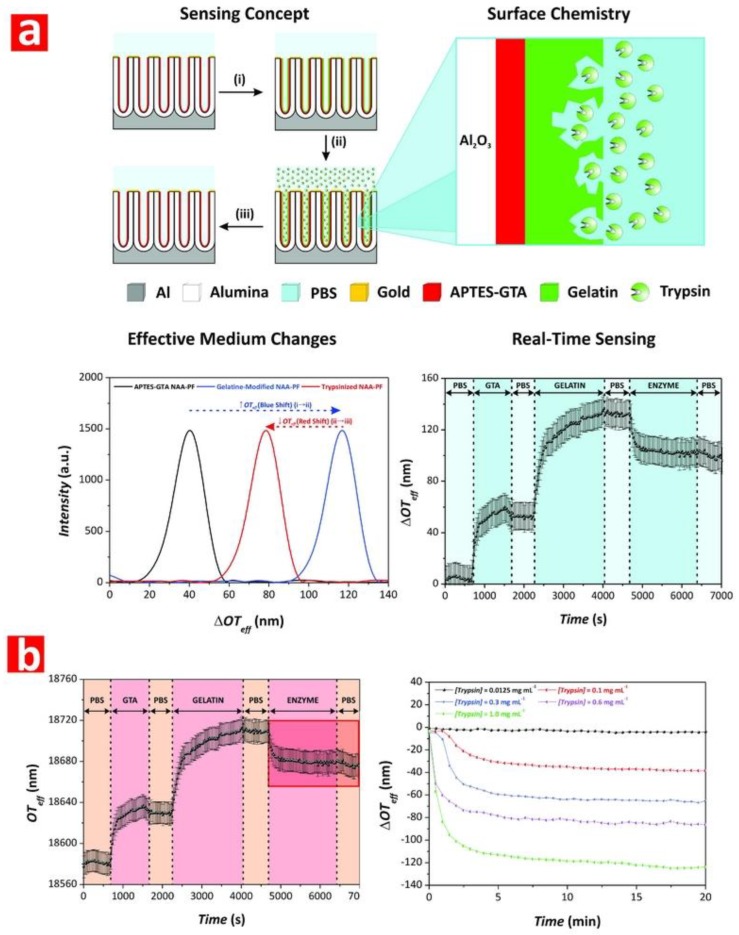
Examples of applicability of NAA-PCs in chemo- and biosensing applications. (**a**) Sensing concept, surface chemistry, and sensing principle for enzymatic sensor combining gelatin-functionalized NAA- Fabry–Perót interferometers (FPIs) and reflectometric interference spectroscopy (RIfS). (**b**) Real-time monitoring of effective optical thickness changes as a function of enzyme concentration. Reproduced from [244], with copyright permission from American Chemical Society, 2015.

Kumeria et al. presented an ultrasensitive NAA-FPI sensor to detect *Au*^3+^ ions in aqueous matrices using RIfS [119]. Chemical selectivity was attained by functionalizing the inner surface of NAA-FPIs with 3-mercaptopropyl-triethoxysilane (MPTES), which contain a thiol functional group with high affinity toward gold (III) ions. Interactions between gold ions and thiol functional groups induced ∆*OT_eff_*, the magnitude of which was found to be dependent on the concentration of Au^3+^. The proposed sensor had a working range from 0.1 to 80 μM with a sensitivity of 1.09 nm μM^−1^ and a *LLoD* of 0.1 μM. The chemical selectivity of the system toward Au^3+^ was verified through a series of selectivity tests with solutions containing potential interfering ions. Furthermore, the system’s performance for real-life applications was demonstrated by detecting Au^3+^ in tap water and phosphate buffer solution. An interferometric sensor based on the combination of gelatin-functionalized NAA-FPIs and RIfS was developed by Nemati et al. for the selective detection of trypsin enzyme (Figure 12a,b) [244].

**Figure 13:**
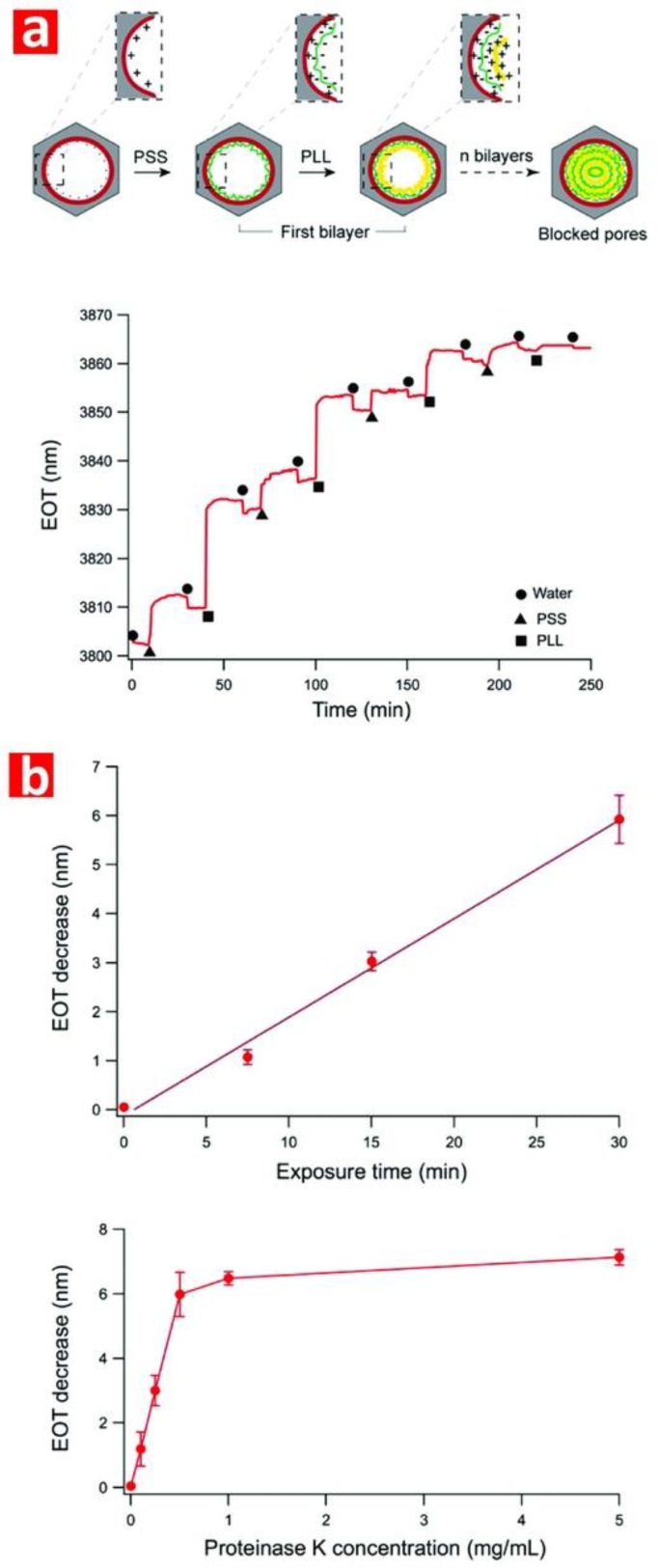
Examples of applicability of NAA-PCs in chemo- and biosensing applications. (**a**) Real-time monitoring of chemical functionalization of NAA-FPIs by polyelectrolyte layers using RIfS. (**b**) Sensing performance of enzymatic sensor as a function of exposure time and concentration upon digestion of polyelectrolyte layers. Reproduced from [142], with copyright permission from American Chemical Society, 2015.

The selectivity, affinity, and specificity of gelatin-modified NAA-FPIs toward trypsin were verified by exposing a set of these PC platforms to nonspecific enzymes (i.e., chymotrypsin and horseradish peroxidase) as well as hemoglobin-modified NAA-PFIs to trypsin. The digestion of gelatin immobilized on the inner surface of NAA by trypsin enzyme was quantified by ∆*OT_eff_* as a function of the concentration of trypsin. This enzymatic sensor achieved a sensitivity of −106.9 nm (mg mL^−1^)^−1^, a *LLoD* of 0.025 mg mL^−1^, and a *R*^2^ of 0.9140. This system enabled the quantification of the kinetics of gelatin digestion (i.e., Michaelis–Menten parameters) by the reaction velocity, defined by ∆*OT_eff_* and reaction time and the concentration of gelatin. Krismastuti et al. developed an enzymatic sensor combining NAA-FPIs and RIfS to detect levels of proteinase K (Figure 13a,b) [142]. The surface chemistry of NAA-FPIs was modified with polyelectrolytes by the LbL deposition technique. The enzymatic degradation of the LbL layers by proteinase K was characterized by ∆*OT_eff_* as a function of exposure time and concentration of proteinase K. The sensitivity of the enzymatic sensor was 12.311 nm (mg mL^−1^)^−1^ with a *LLoD* of 0.06 mg mL^−1^. This sensor was also able to detect the presence of proteinase K in human wound fluid, demonstrating the potential of this system for detection of bacterial infections in chronic wounds.

Lee et al. combined NAA-FPIs as biochip substrate with RIfS to design an optical sensor for quantification of β-galactosidase [245]. NAA-PFIs were functionalized with prolinker A via silane-linking chemistry. Blue shifts in the Fabry–Perót fringe pattern, due to molecular interactions, were translated into ∆*OT_eff_* as a function of β-galactosidase concentration. This protein sensing system had a linear working range from 0.05 to 5 units enzyme mL^−1^ and a sensitivity of 39.04 nm (unit mL^−1^)^−1^. Bae et al. fabricated a hybrid plasmon-coupled NAA-FPIs sensing system, where the sensing platforms were produced by conventional two-step anodization and coated with a layer of gold [246]. This sensing system was able to detect changes in the effective refractive index of NAA-FPIs upon infiltration of Cargille refractive index fluids, achieving a maximum sensitivity of 324 nm RIU^−1^.

Changes in the refractive index of the medium filling the nanopores produced visible shifts in the characteristic interferometric color of NAA-FPIs. Image analysis of these liquid-infiltrated NAA-FPIs was performed in the CIELab 1931 space as a function of Euclidean distance between two color points, which yielded a perceptual refractive index change (i.e., lower limit of colorimetric sensing performance) of 0.006. Santos et al. developed a photoluminescent enzymatic sensor using NAA-FPIs produced by two-step anodization in oxalic acid electrolyte as sensing platforms [247]. Functionalization of the inner surface of nanopores with trypsin was carried out through several stages, which were monitored by the characteristic interferometric PL spectra in terms of number, intensity, and position of PL oscillations.

Each functionalization step yielded a shift in effective optical thickness of these NAA-FPIs obtained from the PL spectra. This methodology allowed accurate detection and quantification of immobilized enzyme within NAA-FPIs. Santos et al. developed a barcode system based on the characteristic PL spectrum of NAA-FPIs, in which shifts in the characteristic interferometric pattern of NAA-FPIs were used as a principle to develop a sensing system [13]. NAA-FPIs were infiltrated with glucose and oxazine dye, which caused red shifts in the position of the characteristic PL oscillations. Shifts in oscillations in the characteristic PL spectrum upon infiltration were translated into exclusive barcodes that characterize these processes. Santos et al. developed a PL-based sensor using NAA-FPI platforms for nonspecific and specific molecular adsorption [43]. Nonspecific interactions of NAA-FPIs with glucose were characterized by effective optical thickness changes measured from the interferometric pattern in the PL spectrum of NAA-FPIs. An analysis of these changes as a function of the concentration of glucose established a sensitivity of 0.013% mM^−1^ and *LLoD* of 0.01 M. The proposed sensor was also used to detect L-cysteine under specific adsorption conditions. The binding of L-cysteine, a model peptide, to silane-modified NAA-FPIs were monitored by ∆*OT_eff_* quantified from the characteristic PL spectrum as a function of the concentration of L-cysteine. Under specific adsorption conditions, the proposed PL sensor had a sensitivity of 0.029% mM^−1^ and *LLoD* of 5 mM for L-cysteine. Ferro et al. combined NAA-FPIs with PL spectroscopy for glucose sensing, where the characteristic PL spectra of NAA-FPIs was monitored after immersion in solutions containing different concentrations of glucose [248]. A multivariate analysis was used to derive a quantitative model for glucose concentration determination and the *LLoD* of the sensing system was 0.01 mol L^−1^. Trivinho-Strixino et al. developed a PL-based sensor using NAA-FPIs to detect commercial pesticide molecules [249]. NAA-PFIs were infiltrated with different concentrations of chloropyriphos pesticide and shifts in the characteristic PL spectra of these PC platforms were quantified. This analysis established a linear red shift of the characteristic PL band with increasing pesticide concentration, enabling the qualitative determination of pesticide.

### 4.5. Other Nanoporous Anodic Alumina Photonic Crystal Sensing Platforms

**Figure 14:**
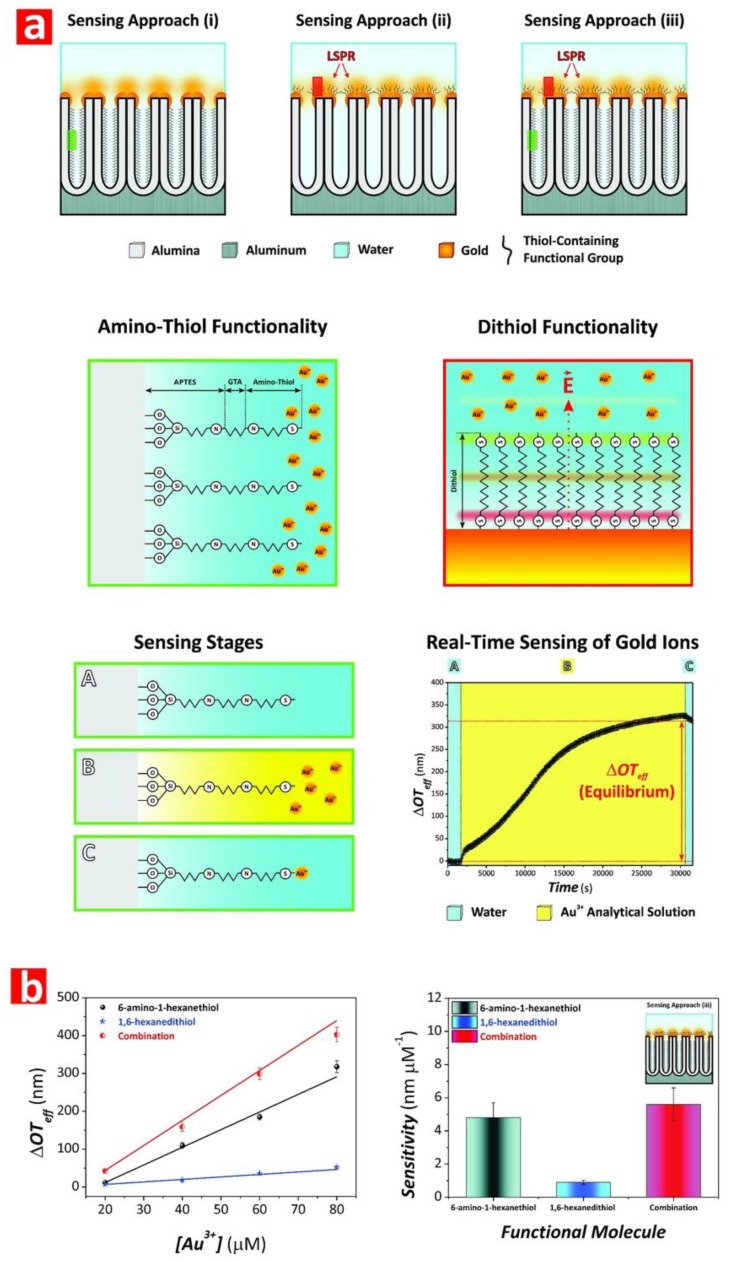
Examples of applicability of NAA-PCs in chemo- and biosensing applications. (**a**) Schematics showing the surface chemistry architecture and sensing concept of gold-modified NAA-FPIs for real-time sensing of gold ions. (**b**) Sensitivity as a function of the surface chemistry architecture in NAA-FPIs. Reproduced from [123], with copyright permission from American Chemical Society, 2017.

Law et al. combined NAA-PC platforms with RIfS to develop a sensing system able to monitor in real-time the formation of self-assembled monolayers of thiol molecules (Figure 14a,b) [123]. NAA-PCs with effective medium modulated in a sawtooth-like fashion were coated with gold and exposed to different concentrations of 11-mercaptoundecanoic acid (11-MUA). The gold–thiol interaction resulted in a shift in the characteristic PSB of these PCs the in RIfS spectra, which was found to red-shift with the concentration of 11-MUA. An analysis of this interaction revealed that it follows a Langmuir isotherm binding model.

The working range of this sensor was from 0.3125 to 1.25 mM: *S* = 8.88 nm mM^−1^, *LLoD* = 0.3125 mM, and *R*^2^ = 0.90. Yan et al. fabricated NAA-PCs with sinusoidally modulated nanopores, the sensitivity of which was assessed by infiltrating their nanoporous structure with analytes of different refractive index such as water, ethyl alcohol, ethylene glycol, and glycerol [250]. The sensitivity of these NAA-PCs was assessed using shifts in the position of the characteristic PSB upon infiltration. Shifts in the position of the PSB were found to follow a linear trend with increasing refractive index of the media filling the nanopores, with a sensitivity of 108.5 nm RIU^−1^. Shang et al. developed NAA-PCs with narrow photonic stopbands produced by a modified two-step anodization process incorporating a step voltage compensation. The application of these PCs as sensing platforms for gas sensing was demonstrated [251]. When NAA-PCs were exposed to an increasing concentration of anhydrous ethanol gas, from 0 to 13.72 mmol L^−1^, the PSB’s position in the transmission spectra underwent a red shift of 66 nm. This red shift was also observed when NAA-PCs were exposed to a saturated ethanol gas environment for longer time. Using the same anodization approach, Shang et al. used these NAA-PCs to monitor the adsorption of organic molecules via capillary condensation [252]. The adsorption of ethanol, methanol, acetone, and toluene in both gas and liquid forms were monitored through shifts in the characteristic PSB of these PC structures. Their observations indicate that, upon saturation of condensed analyte molecules in the nanopores of NAA-PCs, the position of the PSB red-shifts whereas the transmission intensity is reduced.

Table 3 collates the most representative systems combining NAA-PCs with different optical techniques, summarizing the sensing performances of these systems. These studies demonstrate that NAA-PCs are excellent sensing platforms due to their highly controllable and tunable effective medium and versatile surface chemistry, which can be precisely engineered to achieve outstanding performances for a broad range of chemo- and biosensing applications.

## 5. Conclusions

This review provides a comprehensive and up-to-date collation of fundamental and applied developments of nanoporous anodic alumina photonic crystals as optical platforms for chemo- and biosensing applications. Pioneering studies over the past two decades have demonstrated the potential of this nanoporous material to be integrated with different optical techniques to develop sensing systems with unique properties and capabilities. NAA provides an excellent PC platform due to its self-organized and highly controllable nanoporous structure, the optical properties of which can be precisely engineered by the fabrication conditions. The effective medium of NAA-PCs can be modulated in a multidimensional fashion to control light in different ways and utilize light–matter interactions at the nanoscale to achieve high sensitivity for different sensing applications. Furthermore, a broad range of chemical and physical methods can be used to engineer the surface chemistry of NAA-PCs to achieve chemical selectivity toward analytes and molecules of interest. Pioneering studies demonstrated the potential of NAA-PCs for optical applications and recent developments of rationally designed pulse-like anodization strategies have boosted the applicability of multidimensional NAA-PCs for chemo- and biosensing applications, providing new opportunities to develop optical systems based on different sensing principles (e.g., colorimetry, reflection, transmission, photoluminescence, etc.).

Anodization is a cost-competitive, simple, and fully scalable nanofabrication approach that enables the generation of NAA-PCs with finely tuned optical properties across the spectral regions, from UV to IR. However, more systematic theoretical and experimental investigations will be needed to fully develop the potential of this technology. NAA has intrinsic limitations such as its relatively low refractive index and its limited range of lattice constants. The design of novel anodization strategies and new NAA-PC architectures could overcome these intrinsic limitations and enable the development of PC platforms with unprecedented optical properties for sensing applications. Proof-of-concept studies have demonstrated the integration of NAA-PCs into fully functional optical sensing devices and lab-on-a-chip systems. However, more fundamental and applied research will be required to harness the potential of NAA-PC technology and utilize its unique properties to create practical devices and systems with optimal performances for real-world chemo- and biosensing applications. These systems will require the integration of NAA-PCs with other technologies such as microfabrication and microfluidics as well as new functionalization approaches to achieve high chemical selectivity toward analytes of interest.

As this review demonstrates, NAA-PC technology has huge potential and broad applicability for sensing applications. These photonic platform materials are an attractive technology to realize unique sensing concepts and devices that are distinctly different from and complementary to existing technologies such as porous silicon or inverted opals. It is thus expected that this highly dynamic and exciting field will continue to grow and spread toward more sophisticated PC structures, sensing concepts and applications.

## Figures and Tables

**Table 1:** Comparison of properties of self-assembled monolayers (SAMs) of different molecules.

SAM	Advantages	Limitations	Quality
**Alkanoic acid**	- Wide range of carboxylates with different functionalities available - Environmentally friendly - Densely packed SAMs	- Hydrolytically unstable in aqueous media - Relatively easy desorption and exchange	Good
**Organophosphonates**	- Stable and highly resistant to hydrolysis - Densely packed SAMs	- Potential precipitation of phosphonates	Excellent
**Organosilanes**	- Rapid covalent binding with substrates - Further chemical modification without compromising the integrity of SAMs	- Difficult to obtain uniform SAMs with limited packing density - High reactivity - Hydrolytically unstable under aqueous and humid conditions - Limited reproducibility	Excellent
**Organosulfurs**	- Simple and well-established self-assembly process	- Prone to oxidation upon air exposure - Rapid decomposition at high temperature - Undergo displacement when exposed to adsorbates - Conformational defects and cross-contamination	Good
**Alkenes and alkynes**	- Good stability in water and high temperature - Ease of further (bio)functionalization	- Disordered SAMs - Low surface coverage due to poor reaction kinetics	Excellent

**Table 2:** Characteristics and optical properties of representative NAA-PCs.

NAA-PC	Anodization *	Photonic Features	Refs. **
**NAA-FPI**	- One-step anodization - Two-step anodization	- Interference fringes (out-of-plane) - PSB (in-plane)	[43] [46,47,48,49] [111] [119] [125] [142] [240,241,242,243,244] [244]
**NAA-μCV**	- STPA with constant step between mirrors - SPA with constant step between mirrors - SATPA with constant step between mirrors - PSTPA with constant step, progressive variation of electrolyte temperature, or phase shift between mirrors	- Resonance band within PSB	[82,83,84,85] [239]
**NAA-DBR**	- STPA - PSTPA	- Broad PSB	[75] [81] [110,111] [231,232] [234,235]
**NAA-GIF**	- SPA - PSTPA	- Narrow PSB	[77] [80] [112] [171] [238]
**Apo-NAA-DBR**	- ASTPA	- Narrow PSB	[80] [85] [233]
**Apo-NAA-GIF**	- ASPA	- Ultra-narrow PSB	[78,79,80,81]
**3D NAA-PC (DBR)**	- SPA with final etching	- Broad PSB (in-plane and out-of-plane)	[75,76]
**NAA-BPFs**	- STPA - PSTPA - STPA + SPA	- Versatile PSB or PSB across the spectral regions	[79] [86]
**NAA-LBPFs**	- SPA with asymmetric etching	- Narrow PSB with position variable across the surface	[87]

* STPA = stepwise pulse anodization; SPA = sinusoidal pulse anodization; SATPA = sawtooth pulse anodization; PSTPA = pseudo-stepwise pulse anodization; ASTPA = apodized stepwise pulse anodization; ASPA = apodized sinusoidal pulse anodization. ** Representative references.

**Table 3:** Comparison of the sensing performance for representative optical sensing system using NAA-PCs.

Ref.	NAA-PC	Analyte	Sensing Technique *	*S*	*LLoD*
[231]	NAA-DBR	Au^3+^	RIfS	22.2 nm µM^−1^	0.16 µM
[232]	NAA-DBR	Vitamin C	RIfS	227 nm µM^−1^	0.02 µM
[111]	NAA-DBR	Hg^2+^	RIfS	0.0115 nm µM^−1^	4.20 µM
[234]	NAA-DBR	Alkanes	TS	71.4 nm RIU^−1^	-
[234]	NAA-DBR	Alcohols	TS	61.9 nm RIU^−1^	-
[238]	NAA-GIF	D-glucose	RIfS	4.93 nm M^−1^	0.01 M
[153]	NAA-GIF	Indomethacin	RIfS	0.63 nm mM^−1^	65 µM
[172]	NAA-GIF	Indomethacin	RIfS	89.8 nm mM^−1^	
[172]	NAA-GIF	Sulfadymethoxine	RIfS	5.50 nm mM^−1^	-
[172]	NAA-GIF	Warfarin	RIfS	18.4 nm mM^−1^	-
[172]	NAA-GIF	Coumarin	RIfS	8.30 nm mM^−1^	-
[172]	NAA-GIF	Salicylic acid	RIfS	4.90 nm mM^−1^	-
[112]	NAA-GIF	Hg^2+^	RIfS	0.072 nm µM^−1^	1 µM
[81]	NAA-GIF	Polar and nonpolar analytes	RS	441 nm RIU^−1^	-
[83]	NAA-μCVs	Au^3+^	RIfS	8.88 nm mM^−1^	0.3125 mM
[111]	NAA-FPI	Glucose	RIfS	18.42% RIU^−1^	0.084 RIU
[111]	NAA-FPI	Hg^2+^	RIfS	0.0009% µM^−1^	22.82 RIU
[123]	NAA-FPI	Au^3+^	RIfS	5.6 nm µM^−1^	
[240]	NAA-FPI	TNF-α	RIfS	22.384 nm (ng mL^−1^)^−1^	0.13 μg mL^−1^
[241]	NAA-FPI	Circulating tumour cells	RIfS	-	1000 cells mL^−1^
[232]	NAA-FPI	D-glucose	RIfS	0.007% mM^−1^	100 mM
[43]	NAA-FPI	L-cysteine	RIfS	0.026% mM^−1^	5 mM
[113]	NAA-FPI	Au^3+^	RIfS	1.09 nm µM^−1^	0.1 µM
[244]	NAA-FPI	Tyrypsin	RIfS	−106.9 nm (mg mL^−1^)^−1^	0.025 mg mL^−1^
[142]	NAA-FPI	Proteinase K	RIfS	12.311 nm (mg mL^−1^)^−1^	0.06 mg mL^−1^
[245]	NAA-FPI	β-galactosidase	RIfS	39.04 nm (unit mL^−1^)^−1^	0.05 units enzyme mL^−1^
[43]	NAA-FPI	D-glucose	PL	0.013% mM^−1^	0.01 M
[43]	NAA-FPI	L-cysteine	PL	0.029% mM^−1^	5 mM

* RIfS = reflectometric interference spectroscopy, TS = transmission spectroscopy, RS = reflection spectroscopy, and PL = photoluminescence spectroscopy.
